# CNS inflammatory demyelinating events after COVID-19 vaccines: A case series and systematic review

**DOI:** 10.3389/fneur.2022.1018785

**Published:** 2022-12-01

**Authors:** Virginia Rinaldi, Gianmarco Bellucci, Maria Chiara Buscarinu, Roberta Reniè, Antonio Marrone, Martina Nasello, Valeria Zancan, Riccardo Nistri, Roberto Palumbo, Antonio Salerno, Marco Salvetti, Giovanni Ristori

**Affiliations:** ^1^Neurology Unit, Department of Neurosciences, Mental Health and Sensory Organs (NESMOS), Sapienza University of Rome, Rome, Italy; ^2^Department of Neurosciences, Sapienza University of Rome, Rome, Italy; ^3^Neurology Unit, San Giovanni Addolorata Hospital, Rome, Italy; ^4^IRCCS Istituto Neurologico Mediterraneo Neuromed, Pozzilli, Italy; ^5^Neuroimmunology Unit, IRCCS Fondazione Santa Lucia, Rome, Italy

**Keywords:** ADEM, transverse myelitis, multiple sclerosis, NMOSD, MOGAD, COVID-19, vaccines

## Abstract

**Background:**

Vaccinations provided the most effective tool to fight the SARS-CoV-2 pandemic. It is now well established that COVID-19 vaccines are safe for the general population; however, some cases of rare adverse events following immunization have been described, including CNS Inflammatory Demyelinating Events (CIDEs). Although observational studies are showing that these events are rare and vaccines' benefits highly outweigh the risks, collecting and characterizing post-COVID-19 vaccine CIDEs might be relevant to single out potential risk factors and suggest possible underlying mechanisms.

**Methods:**

Here we describe six CIDEs, including two acute transverse myelitis (ATM), three multiple sclerosis (MS), and one neuromyelitis optica spectrum disorder (NMOSD), occurring between 8 and 35 days from a COVID-19 vaccine. Moreover, we performed a systematic literature search of post-COVID-19 vaccines CIDEs, including ATM, ADEM, MS, and NMOSD/MOGAD, published worldwide between December 2020 and December 2021, during 1 year of the vaccination campaign. Clinical/MRI and CSF/serum characteristics were extracted from reviewed studies and pooled-analyzed.

**Results:**

Forty-nine studies were included in the systematic review, reporting a total amount of 85 CIDEs. Considering our additional six cases, 91 CIDEs were summarized, including 24 ATM, 11 ADEM, 47 MS, and nine NMOSD/MOGAD. Overall, CIDEs occurred after both mRNA (*n* = 46), adenoviral-vectored (*n* = 37), and inactivated vaccines (*n* = 8). Adenoviral-vectored vaccines accounted for the majority of ADEM (55%) and NMOSD/MOGAD (56%), while mRNA vaccines were more frequent in MS new diagnoses (87%) and relapses (56%). Age was heterogeneous (19–88) and the female sex was prevalent. Time from vaccine to symptoms onset was notably variable: ADEM and NMOSD/MOGAD had a longer median time of onset (12.5 and 10 days) compared to ATM and MS (6 and 7 days) and further timing differences were observed between events following different vaccine types, with ATM and MS after mRNA-vaccines occurring earlier than those following adenoviral-vectored ones.

**Conclusion:**

Both the prevalence of vaccine types for certain CIDEs and the heterogeneity in time of onset suggest that different mechanisms—with distinct dynamic/kinetic—might underly these events. While epidemiological studies have assessed the safety of COVID-19 vaccines, descriptions and pooled analyses of sporadic cases may still be valuable to gain insights into CIDE's pathophysiology.

## Introduction

The Coronavirus disease 19 (COVID-19) vaccination campaign has no precedent in history for magnitude and speed. Randomized Control Trials (1–4) (RCTs) and real-world studies (5, 6) provided clear-cut evidence of vaccines' effectiveness in reducing infections, severe COVID-19, and deaths, resulting as the major tool to fight the pandemic. Up to now, 40 COVID-19 vaccines have been approved for emergency use by at least one regulatory authority (7) and several are under development (8). These include mRNA/DNA, adenoviral-vectored, protein-based, and whole virus inactivated/live attenuated formulations. Currently, the four vaccines licensed for use in the highest number of countries are the mRNA-based BNT162b2 (Pfizer/BioNTech) and mRNA-1273 (Moderna), and the adenoviral-vectored ChAdOx1 nCoV-19 (Vaxzeviria) and Ad26.COV2.S (Janssen). Phase 3 RCTs have demonstrated their safety in the general population (1–4). However, as the global vaccination campaign advances, data are being collected for Rare Adverse Events (RAEs), negligible from a statistical viewpoint but potentially helpful to suggest candidate risk factors and possible underlying mechanisms. Among RAEs, some cases of CNS Inflammatory Demyelinating Events (CIDEs) following COVID-19 vaccines have been described in literature from December 2020 (9), including both acute syndromes, such as acute transverse myelitis (ATM) and Acute demyelinating encephalomyelitis (ADEM), and relapses of chronic CNS inflammatory demyelinating diseases, such as multiple sclerosis (MS), neuromyelitis optica spectrum disorder (NMOSD), and myelin oligodendrocyte glycoprotein antibody-associated disease (MOGAD). Although an increasing amount of studies are showing that these events are rare and COVID-19 vaccine benefits highly outweigh the risks (10–13), collecting and characterizing post-COVID-19 vaccine CIDEs might still be relevant to gain insights about CNS inflammatory demyelinating diseases pathophysiology.

Here we report six CIDEs occurring after COVID-19 vaccines and present the results of a systematic review and pooled descriptive analysis of an additional 85, published worldwide from 1 December 2020 to 31 December 2021, during 1 year of the vaccination campaign.

## Case series

### Case 1

**Figure 1:**
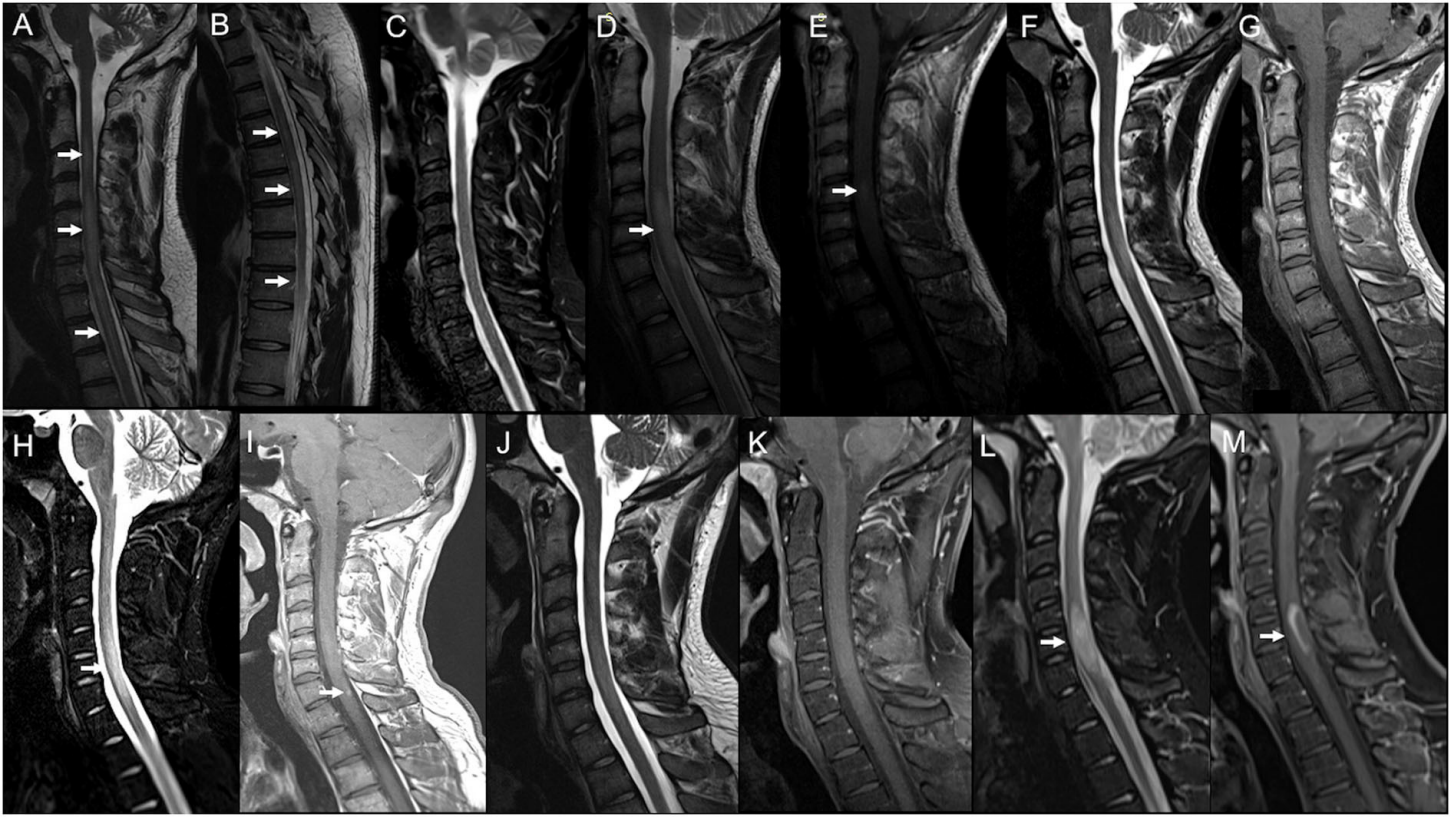
Serial spinal cord MRI scans in Case 1 **(A–C)** and Case 2 **(D–M)**. Case 1: Cervical **(A)** and thoracic **(B)** spinal cord MRI obtained 10 days after Ad26.COV2.S first dose showed a T2-weighted hyperintensity irregularly extending from C3 to medullaris conus with swelling from C4 to C7 (arrows) and no gadolinium enhancement (not shown). **(C)** 8-months MRI follow-up showing the complete resolution of the swelling and marked amelioration of the signal abnormality, barely visible on Short Tau Inversion Recovery (STIR) images. Case 2: **(D)** Spinal cord MRI performed 3 months after Tdap and IVP vaccine administration, showing a T2-weighted hyperintensity from C5 to C7 (arrow) with swelling and blurred contrast-enhancement on T1-weighted sequence **(E)** (arrow). **(F)** 6-months MRI control showing the cervical lesion shrinkage on T2-weighted sequences and no more contrast-enhancement **(G)**. **(H)** Spinal cord MRI obtained 8 days after the BNT162b2 second dose, showing swelling on STIR (arrow) and gadolinium enhancement [**(I)**; arrow] of the previously detected lesion. **(J)** 4-months MRI showed showing marked reduction of the signal abnormality and no more enhancement on the T1-weighted image **(K)**. **(L)** Spinal cord MRI obtained 62 days after the BNT162b2 third dose, showing a new swelling (arrow) and gadolinium ring enhancement **(M)** of the known cervical lesion.

A 34-year-old man, with unremarkable past medical history, presented with numbness in his arms 8 days after receiving the first dose of Ad26.COV2.S vaccine. His condition worsened in a few days: numbness extended to his trunk and legs, and he progressively developed lower limb weakness and urinary retention. He entered our unit 4 days later. On neurological exam, he had light touch/pin-prick hypoesthesia below C4 level, four limbs weakness, and sphincter disturbances requiring catheterization. A spinal cord MRI showed a T2-weighted hyperintensity irregularly extending from C3 to medullaris conus (appearing swallowing from C4 to C7) with no gadolinium enhancement on T1-weighted images (Figures 1A,B); brain MRI resulted negative. Blood count, erythrocyte sedimentation rate, and C-reactive protein were normal. Cerebrospinal fluid (CSF) analysis revealed marked lymphocytosis (310 leucocytes, 90% mononuclear cells) and a slightly elevated protein level. No infectious agent was detected at CSF PCR testing for extensive infectious panel (Herpesviruses, Enterovirus, Parechovirus, i K1, *Haemophilus influenzae, Listeria monocytogenes, Neisseria meningitidis, Streptococcus agalactiae, Streptococcus pneumoniae*, and *Cryptococcus neoformans/gattii*). CSF immunoelectrophoresis (IEP) showed no oligoclonal bands (OCB). Serum anti-AQP-4 and anti-MOG antibodies were negative, as well as an antibody panel for connective tissue diseases. The patient was administered a 5-day course of high-dose IV methylprednisolone (IVMP) followed by oral tapering. After 2 weeks, he showed an almost complete motor recovery in the arms while lower limb weakness and sphincter disturbances partially improved. He started rehabilitation treatment and after 4 months he reported marked amelioration of leg strength and further recovery of limb hypoesthesia. At 8 months of control, he remained clinically stable and MRI showed the complete resolution of the cervical swelling, with the extended T2-hyperintensity appearing fragmented in multiple shorter lesions, barely visible (Figure 1C); brain MRI was still negative.

### Case 2

A 19-year-old man with a negative past medical history presented with numbness and weakness in his right arm in December 2018, 3 months after receiving Diphtheria/Tetanus/Pertussis (Tdap) and Poliovirus (IVP) vaccine booster doses. Symptoms spontaneously resolved in 2 weeks. Three months later, he underwent a spinal cord MRI showing a T2-weighted hyperintensity from C5 to C7, with swelling and blurred contrast-enhancement (Figures 1D,E); the brain MRI was negative. CSF analysis revealed normal cell count/protein level and IEP showed the presence of three OCBs (pattern II, OCB exclusively in CSF). CSF PCR testing for the extensive infectious panel was positive for Enterovirus, but this result was not considered significant due to the absence of prodromal respiratory/gastroenteric illness and clinical/MRI/CSF findings not suggestive of Enterovirus-related ATM. Serum antibodies panel for infectious diseases (including HIV, Herpesviruses, and Borrelia) was unremarkable as well as anti-AQP-4/MOG and connective tissue diseases antibodies. At 6-months of MRI control the cervical lesion appeared shrunk in volume, with no more contrast-enhancing, and no other lesions were detected in the spinal cord and brain (Figures 1F,G). He remained stable at clinical and radiological follow-ups for the next two years, with the last MRI performed in February 2021. On 3rd June 2021, he received the first dose of the BNT162b2 vaccine and the second dose on 28th June. After 8 days, he underwent a brain and spinal cord MRI follow-up, showing the swelling and gadolinium enhancement of the previously detected cervical lesion (Figures 1H,I). He did not complain of any symptoms, except for a mild right-hand numbness occurring 3 weeks after the second dose and lasting for a few days. At a brain and spinal cord MRI performed 4 months later, the cervical lesion appeared reduced in dimension and did not show enhancement (Figures 1J,K). On 22nd December 2021, the patient received the third dose of BNT162b2. After 35 days, he reported numbness in his right arm followed by his right limb weakness. He was admitted to our neurology unit and a new spinal cord MRI showed the swelling and contrast enhancement of the pre-existing cervical lesion (Figures 1L,M); the brain MRI was still negative. A new lumbar puncture showed normal cell count/protein level with no infectious agent detected at PCR, while IEP revealed again the presence of three OCBs (pattern II). Serum anti-AQP-4/anti-MOG and antibodies panel for connective tissue diseases were still unremarkable. The patient was administrated a 5-day course of high-dose IVMP followed by Oral Corticosteroid (OCS) tapering, showing complete recovery after a week. At 4-months of control, he was clinically stable and did not report relapses. He is currently under clinical/radiological follow-up.

### Case 3

**Figure 2:**
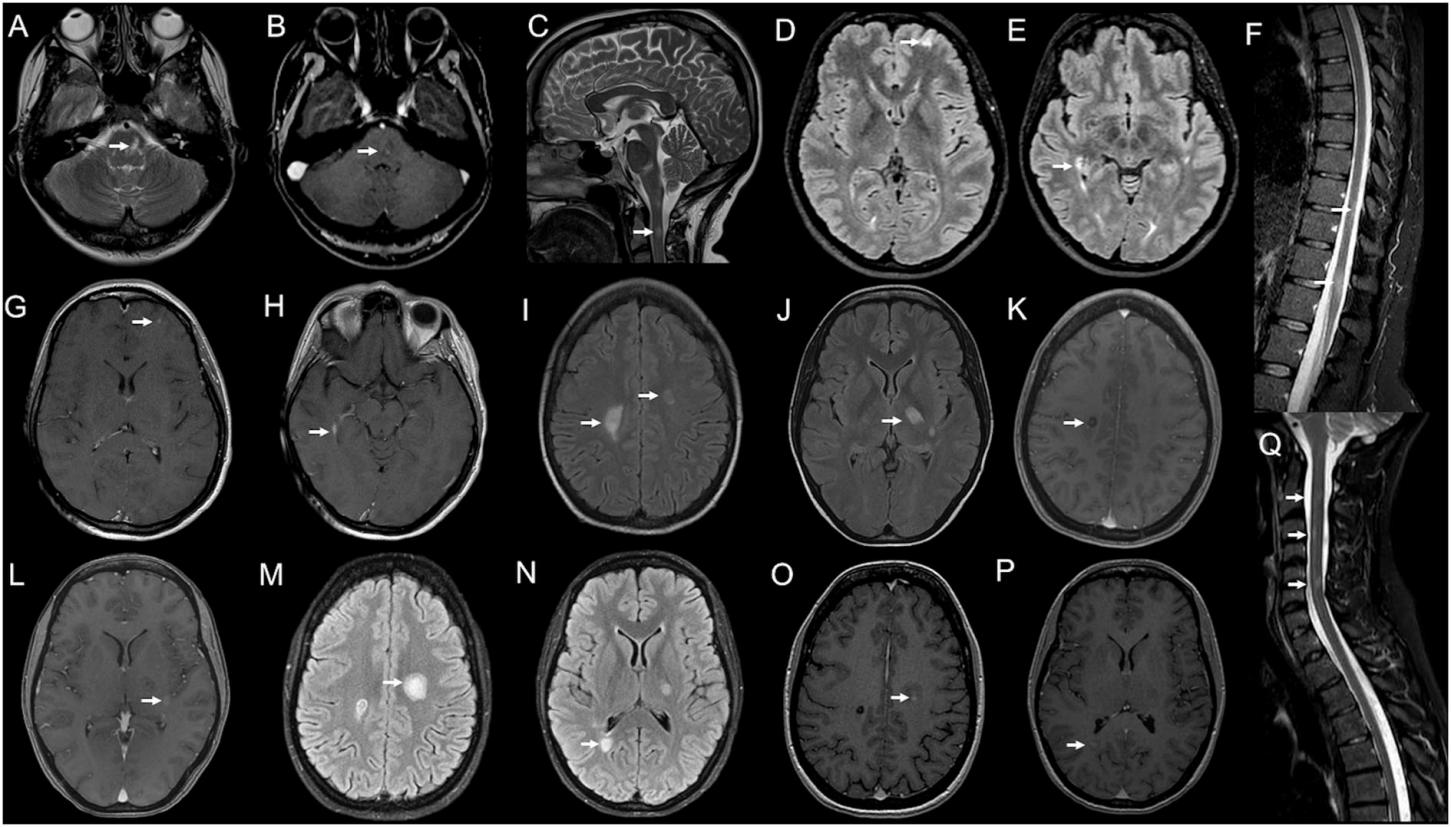
Serial brain and spinal cord MRI scans in Case 3 **(A–H**) and Case 4 **(I–Q)**. Case 3: **(A)** Brain MRI performed 25 days after BNT162b2 second dose, showing a T2-weighted hyperintensity (arrow) in the left paramedian mid-pons with blurred enhancement on T1-weighted images [**(B)**; arrow] and a non-enhancing spinal lesion at C2–C3 level [**(C)**; arrow]. **(D–H)** 2-months MRI follow-up showing two new brain lesions—one juxtacortical in the left frontal hemisphere [**(D)**; arrow] and one periventricular abutting the right lateral ventricle occipital horn [**(E)**; arrow], both with contrast-enhancement [**(G,H)**; arrows] and two spinal lesions at the dorsal level [**(F)**; arrows], with no contrast enhancement (not shown). Case 4: **(J–L)** Brain MRI obtained 10 days after BNT162b2 second dose, revealing T2-weighted hyperintensities in both centra semiovale (CSO) [**(I)**; arrows] and left thalamus/posterior limb of the internal capsule (IC) [**(J)**; arrow]; lesions in the right CSO [**(K)**; arrow] and left IC [**(L)**; arrow] showed blurred contrast enhancement on T1-weighted images. **(M–P)** 20-days MRI control showing the enlargement of the left CSO lesion [**(M)**; arrow] and a new lesion in the right peritrigonal area [**(N)**, arrow], both with gadolinium-enhancement on T1-weighted images [**(O,P)**; arrows]. **(Q)** Spinal cord MRI—obtained 10 days after the brain exam—showing three blurred areas at C2, C3, and C5–C6 levels on STIR sequences, with no enhancement (not shown).

A 40-year-old woman, with a history of renal cell carcinoma and no other comorbidities, received the first dose of the BNT162b2 vaccine on 5th May 2021, and the second dose 5 weeks later. Ten days after the first dose, she presented with numbness in her hands progressively extending to all the upper limbs. On 4th July −25 days after the second dose—she developed diplopia in her left horizontal gaze. She was admitted to the emergency unit and an abduction deficit in her left eye was found. A brain and cervical spinal cord MRI showed a T2-weighted hyperintensity with blurred enhancement in the left paramedian mid-pons (Figures 2A,B) and a non-enhancing cervical spinal lesion at C2–C3 level (Figure 2C). She was administered a 5-day course of high-dose IVMP and almost completely recovered within 1 week. Two months of the brain and spinal cord MRI control showed two new enhancing supratentorial lesions—one juxtacortical in the left frontal hemisphere and one periventricular abutting the right lateral ventricle occipital horn—and two spinal lesions at the dorsal level, with no contrast enhancement (we cannot define their time of onset as the first MRI lacked a dorsal study; Figures 2D–H). Blood counts and C-reactive protein were normal. Anti-AQP-4/anti-MOG and antibodies for connective tissue diseases were negative. Serum panel for infectious diseases was unremarkable, except for Epstein-Barr Virus (EBV) serology resulting positive for anti-EBV VCA IgM (titer 75.3 U/ml), VCA IgG (>750 U/ml), EBNA IgG (239 U/ml), while EBV EA IgG was negative (9 U/ml). CSF analysis revealed normal cell count, protein, and glucose, and no infectious agent (including EBV) was detected at CSF PCR; CSF IEP showed the presence of 12 OCB (pattern II). She was diagnosed with MS and Natalizumab was started.

### Case 4

A 27-year-old woman, with no previous clinical history, received the first dose of BNT162b2 vaccine on 16th June 2021 and the second dose after 21 days. One week later she reported mild weakness in her left leg spontaneously resolving in 10 days. A few days later, she developed a right facio-brachial motor deficit and dysarthria. She was admitted to the emergency unit and a brain MRI revealed T2-weighted hyperintensities in both centra semiovale (CSO) and left thalamus/posterior limb of the internal capsule (IC); lesions in the right CSO and left IC showed blurred contrast enhancement (Figures 2I–L); spinal cord MRI was negative. CSF analysis revealed normal cell count/protein level and a negative extensive infectious panel, while IEP showed the presence of OCB (pattern II). Serum anti-AQP-4/anti-MOG antibodies resulted negative as well as connective tissue diseases panel except for the positivity of anti-nuclear antibodies (titer 1:160), considered a non-specific finding. Visual evoked potentials did not show abnormalities. She was administrated a 5-day course of high-dose IVMP, with complete recovery. One week later, she complained of right side weakness again—this time involving her leg also—and dysarthria. She started assuming OCS with no improvement. A new brain MRI revealed a new active lesion in the right peritrigonal area and an increase in the size of the previously detected lesions in the left CSO—this time gadolinium-enhancing—whereas areas in right CSO and left IC showed no more enhancement (Figures 2M–P). A spinal cord MRI—performed 10 days after—revealed the presence of three not enhancing cervical areas (at C2, C3, and C5–C6 levels; Figure 2Q). She was diagnosed with MS, and a second 5-day course of high-dose IVMP followed by OCS tapering was administrated. Poor clinical response was obtained and five plasma exchange (PEX) sessions were performed, with a resolution of dysarthria and slight recovery of right hemiparesis. At 2-months of MRI control, the left CSO and right peritrigonal lesions were still contrast-enhancing and a 3-day course of high-dose IVMP was administrated. She promptly started physical therapy with marked improvement in motor performance. She received the first cycle of Cladribine in October 2021. At the three-month follow-up, she almost fully recovered, only showing right-side brisk reflexes and mild oscillations at the position test in her right leg [Expanded Disability Status Scale (EDSS) score 1].

### Case 5

**Figure 3:**
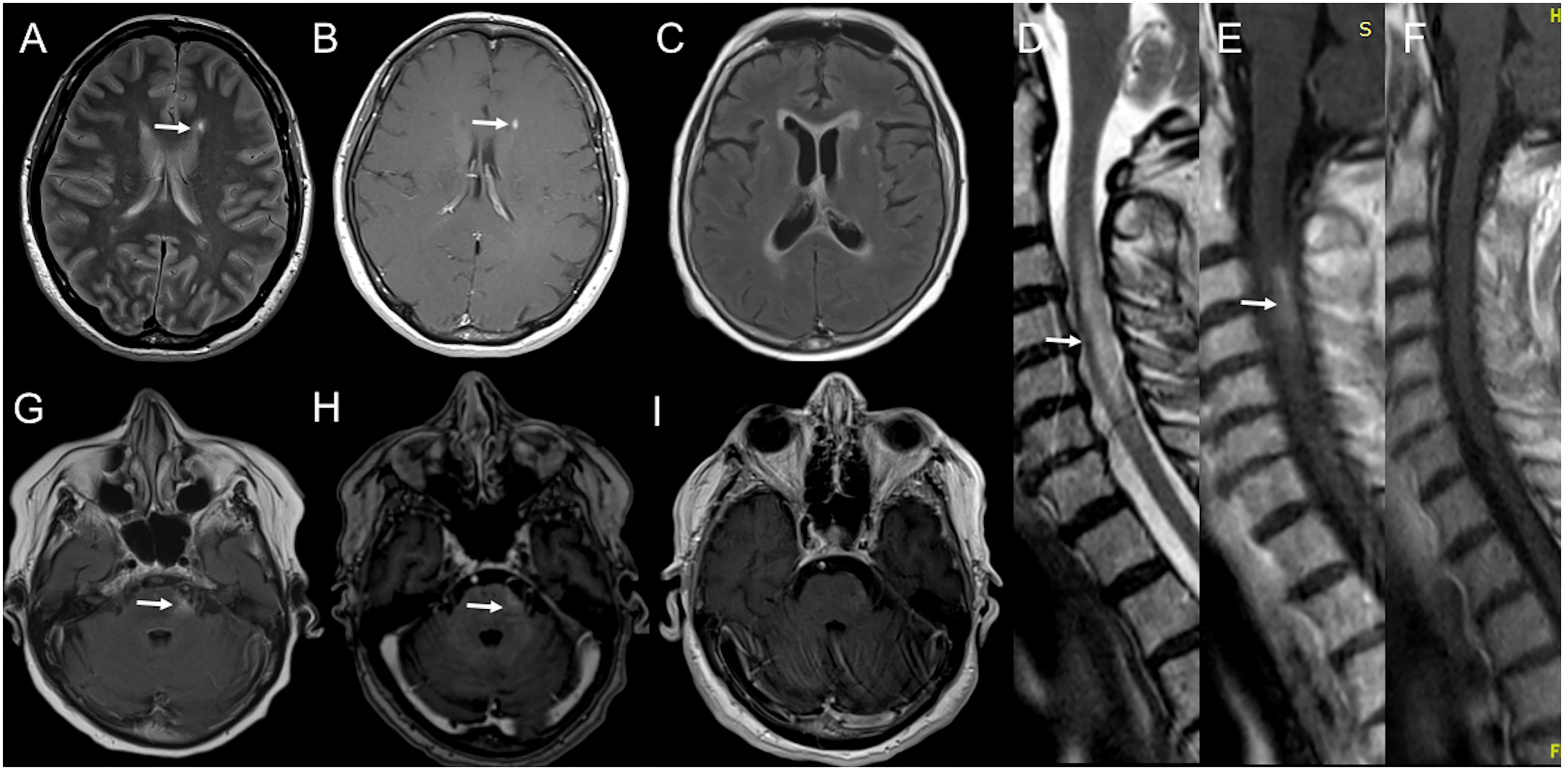
Serial brain and spinal cord MRI scans in Case 5 **(A,B)** and Case 6 **(C–I)**. Case 5: **(A)** Brain MRI performed 28 days after BNT162b2 first dose, showing a new left lateral periventricular T2-weighted hyperintensity (arrow) with contrast enhancement [**(B)**; arrow]. Case 6: **(D)** Spinal cord MRI obtained 12 days after BNT162b2 second dose, showing a T2-weighted hyperintensity from C2 to C7 with swelling (arrow) and gadolinium enhancement [**(E)**; arrow]. **(G)** Brain MRI performed few days later showed a T2-weighted hyperintensity in left lateral pons (arrow) with blurred contrast enhancement [**(H)**; arrow] and confluent supratentorial bilateral periventricular hyperintensities on FLAIR images **(C)**. **(I)** 7-days MRI controls revealed the complete resolution of gadolinium enhancement of the left pontine lesion and the volume shrinkage of the cervical lesion with no more contrast enhancement on T1-weighted images **(F)**.

A 53-year-old woman was diagnosed with MS at age 32 when she presented with paresthesia to her left limbs and a brain/spinal cord MRI showed dissemination in space and time. She was placed on interferon beta-1a with clinical and radiological stability over the next 10 years. In 2010, she developed numbness in her right arm and a new enhancing cervical lesion was detected in a spinal cord MRI. She was switched to Fingolimod with no evidence of disease activity until 2014 when she reported dysesthesia in her left face and a brain MRI revealed a new active lesion in the left frontal lobe. She was administrated a 5-day course of high-dose IVMP with full recovery. Since then, she remained stable at an EDSS of 2.5 at 6-month clinical controls and annual MRI follow-up. On 31st May 2021, she received the first dose of BNT162b2 vaccine and the second dose 35 days later. Two weeks after the first dose administration, she complained of gait imbalance and marked fatigue. A brain/spinal cord MRI—performed 14 days after symptoms onset—revealed a new left lateral periventricular T2-weighted hyperintensity with contrast enhancement (Figures 3A,B). Compared to the previous neurological exam, she showed moderate gait ataxia leading to an EDSS increase by 1 point. She was administrated a 5-day course of IVMP and clinically improved in a few weeks. At 6-months of control, she reported complete recovery (with EDSS returned to baseline) and the resolution of the new lesion enhancement at MRI. She continued with her current disease-modifying therapy (DMT).

### Case 6

A 75-year-old woman presented with right optic neuritis at age 60, partially resolved after OCS administration. Five years later she developed bilateral optic neuritis, treated with OCS with almost complete recovery. No brain/spinal cord MRI was performed at that time, and she started assuming chronic OCS with clinical stability over the following years. She developed Diabetes Mellitus and osteoporosis, complicated by a lumbar vertebral fracture for which she underwent surgical fixation in May 2021. Since then, she started using the right unilateral assistance in walking. On 3rd June 2021, she received the first dose of BNT162b2 vaccine and the second dose on 1st July. After 10 days, she developed dysesthesia and weakness in her right upper limb, followed by weakness in her legs. She was admitted to our emergency unit. On exam, she had four limbs weakness (with motor strength at MRC scale of grade 2/5 and 3/5 in her upper, and lower limbs, respectively), brisk osteotendinous reflexes, bilateral Babinski sign, and light touch hypoesthesia below D4 level. A spinal cord MRI showed a T2-weighted hyperintensity from C2 to C7 with swelling and gadolinium enhancement on T1-weighted images (Figures 3D,E). A brain MRI performed a few days later showed a T2-weighted hyperintensity in left lateral pons with blurred contrast enhancement and confluent supratentorial bilateral periventricular areas on FLAIR images (Figures 3C,G,H). Blood count, erythrocyte sedimentation rate, and C-reactive protein were normal. CSF analysis revealed normal cell count and protein level. No infectious agent was detected at CSF PCR, while IEP showed the presence of three OCBs (pattern II). Anti-AQP-4 antibodies were positive. Extensive serum panel for infectious diseases was unremarkable, as well as anti-connective tissue and anti-neural surface/onconeural antigens antibodies. Patient was administrated a 5-day course of high-dose IVMP. After 10 days, she showed improvement in limb motor performance, with strength at the MRC scale of grade 4/5 in all limbs. A new MRI revealed the complete resolution of enhancement in the left pontine lesion (Figure 3I) and the cervical area—also appeared significantly shrunk in volume (Figure 3F). The patient was planned to start physical therapy and a DMT (Rituximab/Eculizumab).

## Systematic review

### Methods

A systematic review was performed according to the Preferred Reporting Items for Systematic Reviews and Meta-analysis (PRISMA) guidelines. Data were collected from PubMed, SCOPUS, and Google Scholar databases, considering records published from 1st December 2020 to 31st December 2021. Two reviewers (VR and GB) independently conducted the search using the following relevant medical subject headings (MeSH) and keywords: “myelitis,” “encephalomyelitis,” “multiple sclerosis,” “neuromyelitis optica,” “MOGAD,” “COVID-19,” “SARS-CoV-2,” and “vaccine.” After duplicates' removal, records were screened and selected for full-text assessment according to the following inclusion criteria: (i) records reporting a CNS Inflammatory Demyelinating Event (CIDE) occurred after a COVID-19 vaccine (ii) CIDE was either an acute CNS inflammatory demyelinating syndrome—including ATM and ADEM—or a new diagnosis/relapse of a chronic CNS inflammatory demyelinating disease—including MS, NMOSD, and MOGAD. Additional relevant articles that were referenced in the included studies were hand-searched and underwent the screening process. Among eligible records, studies that were not peer-reviewed and not published in English were ruled out. Reviews, viewpoints, letters, and commentaries—unless reporting a case report—were not considered. Furthermore, studies that did not provide individual data or were not supported by positive MRI findings were also excluded. Once identified, included studies were full-text assessed and the following variables were extracted using a standardized form: authors and country of publication, subjects' age, gender, and past medical history, disease characteristics for patients with previously diagnosed MS, NMOSD, and MOGAD (including clinical disease phenotype and duration, time since last clinical/radiological relapse, most recent EDSS score and treatment with DMT), COVID-19 vaccine type and dose administered, time from vaccine to neurological symptoms onset, CIDE clinical presentation, MRI and CSF/serum analysis findings, administered treatment and recovery outcome. Cases were defined as ATM, ADEM, MS, NMOSD, or MOGAD according to the most recent relative diagnostic criteria (14–18). For MS, NMOSD, and MOGAD, it was specified whether the event led to a new disease diagnosis or consisted of a relapse of a previously defined disease.

Pooled descriptive analysis was performed considering data from both cases reported in the literature and described in our case series. Data were summarized using frequencies/proportions for categorical variables and median/Interquartile Range (IQR)/range for continuous variables. Statistical analysis was conducted using R and RStudio.

### Results

A systematic search identified an initial amount of 851 records, of which, 549 resulted unique after duplicate removal (Supplementary Figure 1). Among them, 455 studies did not meet inclusion criteria. The remaining 94 articles underwent a full-text assessment and 45 records were ruled out according to exclusion criteria. Forty-nine studies were finally included in the systematic review. These accounted for 40 case reports and nine case series, reporting a total number of 85 CIDEs, published in 20 countries worldwide. Considering the additional six cases described in our case series, a total of 91 CIDEs were summarized, including 24 ATM, 11 ADEM, 47 MS (15 new diagnoses and 32 relapses), eight NMOSD (seven new diagnoses and one relapse), and one MOGAD. Data extracted from single cases are reported in Supplementary Tables 1–4. Cases characteristics resulting from the pooled analysis are summarized in Table 1 for acute syndromes (ATM and ADEM) and Table 2 for chronic inflammatory demyelinating diseases (MS and NMOSD/MOGAD).

**Table 1:** Characteristics of ATM and ADEM after COVID-19 vaccines.

	**ATM (*n* = 24)**	**ADEM (*n* = 11)**
**Age** median years (range)	52 (19–85)	46 (19–88)
**Female sex** *n* (%)	12 (50)	8 (73)
**History of IMD** *n* (%)^*^	3 (14)	4 (40)
**Vaccine type** *n* (%)	- mRNA: 10 (42) - AV: 11 (46) - Inactivated: 3 (12)	- mRNA: 3 (27) - AV: 6 (55) - Inactivated: 2 (18)
**Time from vaccine to symptoms onset**^**a**^ median days (range)	6 (1–35) - mRNA: 2.5 (2–3) - AV: 8 (7.5–11) - Inactivated: 5 (5–21)	12.5 (2–30) - mRNA: 14 (13–29) - AV: 9 (2–12) - Inactivated: 22 (14–30)
**Number of very early onset events**^**b**^ *n* (%)	9 - mRNA: 8 (89) - AV: 1 (11) - Inactivated: –	1 - mRNA: – - AV: 1 - Inactivated: –
**MRI**^**c**^ *n* (%)	STM: 7 (29) LETM: 17 (71)	Brain: - Supratentorial: 9 (82) - Infratentorial: 4 (36) Spinal cord: - STM: 1 (9) - LETM: 2 (18)
**CSF**^**d**^ *n* (%)^*^	Pleocytosis: 11 (48) ↑ protein level: 14 (8) OCB presence: 5 (29)	Pleocytosis: 5 (56) ↑ protein level: 1 (20) OCB presence: 1 (10)
**Treatment** *n* (%)	IVMP/OCS: 23 (96) PEX/IVIG: 8 (33)	IVMP/OCS: 10 (91) PEX/IVIG: 5 (46)
**Recovery**^**e**^ *n* (%)	Complete/almost: 10 (42) Partial: 13 (54) Death: 1 (4)	Complete/almost: 6 (55) Partial: 3 (27) Death: 2 (18)

AV, adenoviral-vectored; DMT, disease modifying treatment; IMD, immune-mediated disease; IQR, interquartile range; IVIG, intravenous immunoglobulin; IVMP, high dose intravenous methylprednisolone; n/a, not available data; LETM, longitudinally extensive transverse myelitis; Mab, monoclonal antibody; OCB, oligoclonal bands; OCS, oral corticosteroids; PEX, plasma exchange; STM, short-segment transverse myelitis; TM, transverse myelitis.

^*^Proportions are based on cases with related data available.

^a^Timeframe between vaccine administration and onset of ATM/ADEM symptoms.

^b^Events occurred within 3 days of vaccine administration.

^c^Number of cases presenting different locations/extensions of the brain and/or spinal cord lesions at MRI.

^d^Number of cases presenting CSF-positive findings. CSF pleocytosis and increased protein level were defined as CSF WBC >5/μl and protein level >45 mg/dl, respectively.

^e^Recovery at the last available follow-up.

**Table 2:** Characteristics of MS and NMOSD/MOGAD after COVID-19 vaccines.

	**MS (*****n*** = **47)**		**NMOSD/MOGAD (*n* = 9)**
	**New diagnoses (*n* = 15)**	**Relapses (*n* = 32)**	
**Age** median years (range)	40 (26–36)	39 (22–60)	53 (26–75)
**Female sex** *n* (%)	12 (80)	24 (75)	6 (67)
**Disease duration** median years (IQR)	–	10 (0.25–28)	8^h^
**Time from last relapse**^**a**^ median years (IQR)	–	7 (3–14.25)	n/a
**EDSS**^**b**^ median (range)	n/a	2 (0–6)	n/a
**DMT** *n* (%)	None	Untreated: 10 (31) First-line: 9 (28) Second-line: 13 (41)	Azathioprine^h^
**Vaccine type** *n* (%)	- mRNA: 13 (87) - AV: 2 (13) - Inactivated: –	- mRNA: 18 (56) - AV: 13 (41) - Inactivated: 1 (3)	- mRNA: 2 (22) - AV: 5 (56) - Inactivated: 2 (22)
**Time from vaccine to symptoms onset**^**c**^ median days (range)	7 (1–35) - mRNA: 7 (1–35) - AV: 5.5 (3–8) - Inactivated: –	7 (1–25) - mRNA: 6.5 (3–14) - AV: 10 (7–20) - Inactivated: 2	10 (3–21) - mRNA: 14 (10–18) - AV: 8 (7.5–11) - Inactivated: 6.5 (3–10)
**Number of very early onset events**^**d**^ *n* (%)	6 - mRNA: 5 (83) - AV: 1 (17) - Inactivated: –	9 - mRNA: 6 (67) - AV: 2 (22) - Inactivated: 1 (11)	1 - mRNA: – - AV: – - Inactivated: 1
**MRI**^**e**^ *n* (%)	Brain: - Supratentorial: 12 (80) - Infratentorial: 3 (20) Spinal cord: - STM: 6 (40) - LETM: 0 Optic nerve: 0	Brain: - Supratentorial: 17 (53) - Infratentorial: 7 (22) Spinal cord: - STM: 7 (22) - LETM: 1 (3) Optic nerve: 2 (6)	Brain: - Supratentorial: 3 (33) - Infratentorial: 4 (44) Spinal cord: - STM: 2 (22) - LETM: 4 (44) Optic nerve: 2 (22%)
**CSF**^**f**^ *n* (%)^*^	OCB presence: 12 (92)	n/a	OCB presence: 2 (25)
**Serum**^**f**^ *n* (%)^*^	anti-AQP4: 0 anti-MOG: 0	n/a	anti-AQP4: 6 (75) anti-MOG: 1 (13)
**Treatment** *n* (%)	IVMP/OCS: 15 (100) PEX/IVIG: 3 (20)	IVMP/OCS: 29 (91) PEX/IVIG: 1 (3)	IVMP/OCS: 8 (89) PEX/IVIG: 5 (56)
**Recovery**^**g**^ *n* (%)^*^	Complete/almost: 10 (77) Partial: 3 (23)	Complete/almost: 16 (62) Partial: 10 (39)	Complete/almost: 3 (33) Partial: 6 (67)

AV, adenoviral-vectored; DMT, disease modifying treatment; IQR, interquartile range; IVIG, intravenous immunoglobulin; IVMP, high dose intravenous methylprednisolone; LETM, longitudinally extensive transverse myelitis; Mab, monoclonal antibody; n/a, not available data; OCB, oligoclonal bands; OCS, oral corticosteroids; PEX, plasma exchange; STM, short-segment transverse myelitis; TM, transverse myelitis.

^*^Proportions are based on cases with related data available.

^a^Time from the last clinical and/or radiological relapse.

^b^Expanded Disability Status Scale at baseline.

^c^Timeframe between vaccine administration and onset of MS/NMOSD/MOGAD symptoms.

^d^Events occurred within 3 days of vaccine administration.

^e^Number of cases presenting different location/extension of new T2-weighted/gadolinium-enhancing brain and/or spinal cord lesions at MRI.

^f^Number of cases presenting CSF/serum positive findings.

^g^Recovery at the last available follow-up.

^h^Disease duration and DMT of the unique reported case of NMOSD relapse in a previously diagnosed patient (Case 8, Supplementary Table 4).

#### Acute transverse myelitis

**Figure 4:**
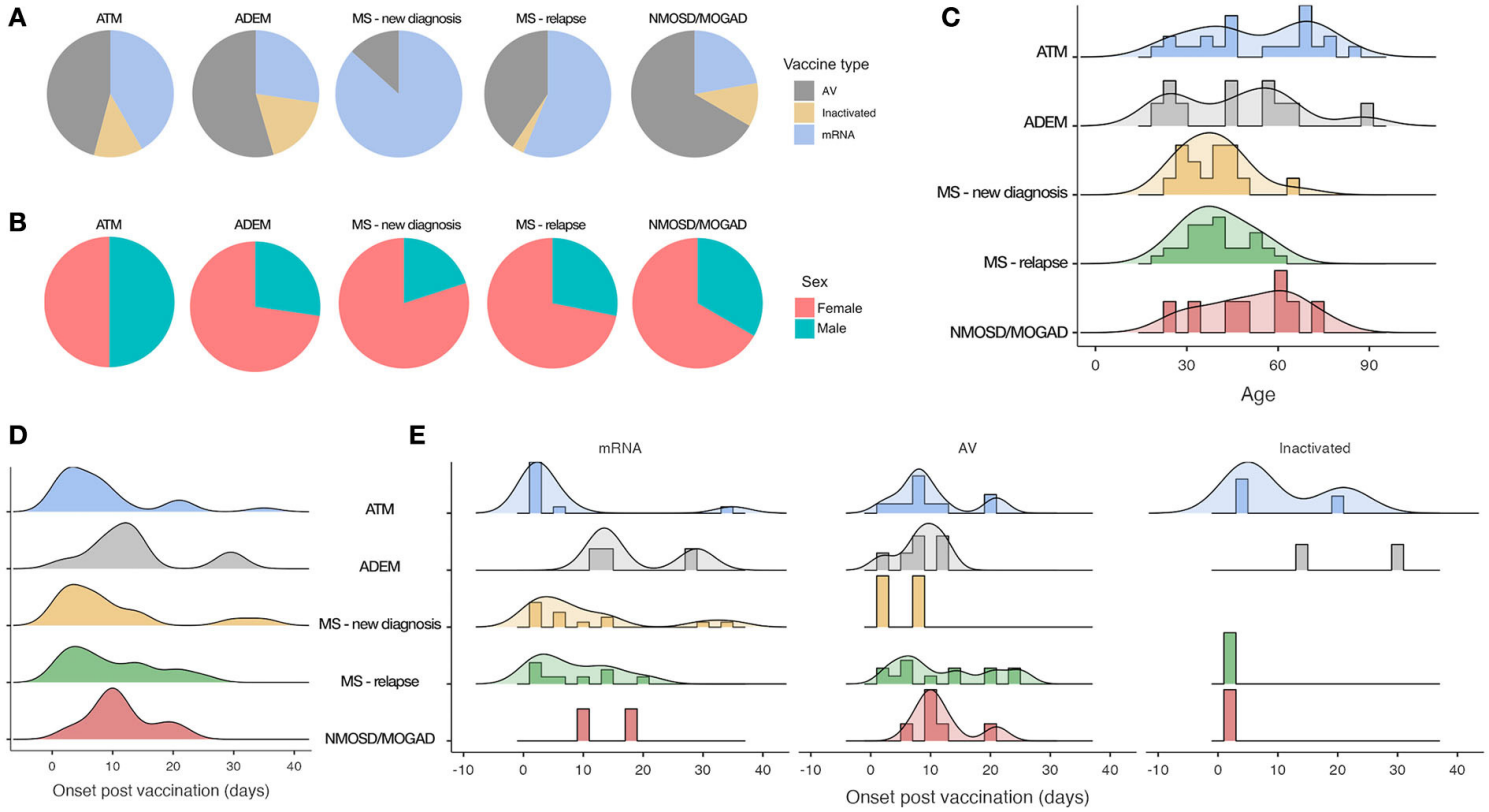
Demographical characteristics and time of onset among different CIDEs. **(A)** Proportions of different vaccine types. **(B)** Proportions of sex categories. **(C)** Age distributions. **(D)** Overall time from vaccine to symptoms onset. **(E)** Time from vaccine to symptoms onset by vaccine type. AV, adenoviral-vectored vaccines.

Among the 24 ATM described (19–40), 11 followed an adenoviral-vectored (46%), 10 an mRNA-based (42%), and three an inactivated vaccine (12%; Figure 4A). Overall, the median time from vaccine to symptoms onset was 6 days (1–35) (Figure 4D), although cases following mRNA-vaccines showed a lower median time (2.5 days, IQR: 2–3) comparing to those after adenoviral-vectored ones (8 days, IQR: 7.5–11; Figure 4E). Moreover, out of nine very early onset ATMs (within 3 days from vaccine administration), eight occurred after an mRNA vaccine. The median age was 52 years and events were equally distributed between sex groups (Figures 4B,C). Three out of 22 reporting cases (14%) had a history of the immune-mediated disease (including atopic dermatitis, asthma, and pulmonary sarcoidosis). All patients presented with a typical clinical syndrome involving sensory, motor, and sphincter systems, while in three cases (Cases 5, 9 and 10; Supplementary Table 1)—all occurring after an adenoviral-vectored vaccine—ATM was also accompanied/followed within 2 weeks by a cranial nerve palsy. Longitudinally Extending Transverse Myelitis (LETM) was the most common MRI finding (71% of cases). CSF OCB resulted absent in 12/17 tested patients, whereas type IV (“mirror,” homologous OCB in CSF and serum) and II (OCB exclusively in CSF) patterns were described in 3 and 2 cases, respectively. All but one patient were treated with a 3–6-day course of high-dose IVMP and a second-line therapy (PEX/IVIG) was administrated in one-third of cases. Complete or almost complete recovery was achieved in 10/24 patients (42%), while others partially recovered, and one patient died of poor general condition.

#### Acute demyelinating encephalomyelitis

Out of 11 ADEM (41–49), six occurred after an adenoviral-vectored (55%), three after an mRNA-based (27%), and two after an inactivated vaccine (18%; Figure 4A). The median time from vaccine to symptoms onset was 12.5 days (2–30) (Figure 4D), with all but one case occurring at least after 7 days. Median age was 46 years and the female sex was prevalent (73%; Figures 4B,C). Four out of 10 reporting cases (40%) had a previous history of immune-mediated disease (including atopic dermatitis, Hashimoto's thyroiditis, polymyalgia rheumatica, and post-infectious rhombencephalitis). Prodromal symptoms (including fever, malaise, headache, and nausea) were common, followed by polyfocal neurological symptoms and/or encephalopathy [defined as decreased level of consciousness/lethargy/behavioral disorders, systemic illness or post-ictal symptoms (15)]. Seizures were reported in three cases. MRI revealed a prominent supratentorial localization with typical -multiple, large, and poorly marginated lesions, whereas the spinal cord was involved in 27% of cases, mostly with a longitudinally extensive feature. CSF OCB was absent in 9/10 tested cases. Acute Hemorrhagic Encephalomyelitis (AHEM) was described in three patients (Cases 3, 4, and 5; Supplementary Table 2), all showing poor outcomes. Complete or almost complete recovery was reached in 6/11 patients (55%) after a 3–7-day course of high-dose IVMP/OCS. Among others, three cases showed partial recovery and two patients died—one presenting an AHEM variant and one due to a rapidly progressive clinical worsening.

#### Multiple sclerosis

Fifteen cases of MS new diagnoses and 32 relapses were described after a COVID-19 vaccine (50–62). Among new diagnoses, 13 occurred after an mRNA vaccine (87%) and two after an adenoviral-vectored one (13%; Figure 4A). Median time from vaccine to symptoms onset was 7 days (1–35) (Figure 4D). Patients were 80% women, with a median age of 40 years (Figures 4B,C). Sensory onset was the most common, followed by pyramidal, cerebellar, truncal, visual, and sphincter systems involvement. MRI showed typical supratentorial lesions (periventricular and/or cortical/iuxtacortical) in the majority of patients, while infratentorial and spinal cord locations were reported in 20 and 40% of cases, respectively. CSF OCB was present in 12/13 tested patients. All were treated with a 3–5 day course of IVMP (with further days of IVMP/PEX needed in just three cases) and recovery was mostly favorable.

Among MS relapses, 18 cases followed an mRNA (56%), 13 an adenoviral-vectored (41%), and 1 an inactivated vaccine (3%; Figure 4A). Overall, median time from vaccine to symptoms onset was 7 days (1–25) (Figure 4D), although relapses following mRNA-vaccines presented with a lower median time [6.5 days (IQR: 3–14)] compared to those after adenoviral-vectored vaccines [10 days (IQR: 7–20); Figure 4E]. Moreover, among nine very early onset events (within 3 days from vaccine administration), six followed an mRNA-vaccine. Median age was 39 years and the female sex was prevalent (Figures 4B,C). In cases reporting disease characteristics, the median EDSS was 2 and 75% had their last clinical and/or radiological relapse at least 3 years before. Ten out of 32 patients (31%) were not taking DMT, while 9/32 (28%) were on first-line DMT, and 13/32 (41%) were on second-line DMT (six of which receiving oral therapy and seven a monoclonal antibody). Active lesions were located exclusively in the brain on 11/28 and involved the spinal cord in 25% of cases. Corticosteroid therapy was performed in the vast majority of patients (followed by PEX in just one case), followed by complete or almost complete recovery in 16/26 reporting cases, while the others partially improved.

#### Neuromyelitis optica spectrum disorder and anti-MOG antibodies-associated disease

Eight cases of NMOSD and one case of MOGAD were reported after a COVID-19 vaccine (50, 58, 63–67). Five followed an adenoviral-vectored (56%), two an mRNA (22%), and two an inactivated vaccine (22%; Figure 4A), with a median time from vaccine to symptoms onset of 10 days (3–21) (Figure 4D). Median age was 53 years, and 6/9 cases were women (Figures 4B,C). Among NMOSD, seven cases were new diagnoses and one consisted in a relapse in a previously diagnosed patient, currently treated with azathioprine (Case 8; Supplementary Table 4). The most frequent core clinical presentation was an acute medullary syndrome, followed by brainstem/area postrema/diencephalic syndromes and optic neuritis. MRI showed typical NMOSD characteristics in all cases (LETM and/or brain peri-ependymal lesions) and anti-AQP4 were positive in all but one patient (Case 2; Supplementary Table 4), who fulfilled seronegative NMOSD diagnostic criteria reporting two clinical cores syndromes with typical MRI features. The unique case of MOGAD was described in a 59-years-old man, presenting an acute medullary syndrome with serum MOG-antibody positivity 13 days after receiving the first dose of ChAdOx1 nCoV-19 (Case 9; Supplementary Table 4). Overall, treatment consisted of IVMP in 8/9 NMOSD/MOGAD patients, followed by PEX in four of them. The outcome was commonly poor, with 6/9 patients not achieving complete recovery.

## Discussion

### Vaccinations and CIDEs

The magnitude and speed of the COVID-19 vaccination campaign allowed us to observe, on a large scale and in an extremely short time period, rare adverse events already described for other vaccines in a more scattered way. Among RAEs, post-vaccination CIDEs are a well-established entity and have been temporally associated with different vaccines. Karussis and Petrou (68) reported 71 cases of CIDEs published in literature from 1979 to 2013, including cases of ADEM, ATM, optic neuritis, MS, and NMOSD The most commonly associated vaccines included influenza, human papillomavirus (HPV), hepatitis A or B, rabies, measles, rubella, yellow fever, anthrax, meningococcus, and tetanus. However, apart from rare exceptions [e.g., ATM following the live attenuated Oral Poliovirus Vaccine (OPV) (69)], a causal link between vaccinations and CIDEs has not been formally confirmed and the association only relies on temporal relation (70).

### CIDEs after COVID-19 vaccines

#### RCTs and observational studies

Phase 3 RCTs for COVID-19 mRNA vaccines—the first to be approved in December 2020—did not report CIDEs during the study period (1, 2), as well as RCT for the adenoviral-vectored Ad26.COV2.S (4). Interim analysis of the four RCTs for ChAdOx1 nCoV-19 reported three cases of ATM among 11,636 participants, two occurring in the treatment group and one in the control (meningococcal) arm (3). Among the first two, one case presented 14 days after the second dose and, although initially regarded as possibly related to vaccination, was eventually diagnosed as an idiopathic ATM. The second case occurred 10 days after the first dose and was instead considered to be related to a pre-existing—but not recognized—MS.

However, RCTs are hardly able to detect RAEs—for insufficient statistical power—and provide only limited information about population subgroups not included in trial protocols, such as persons with autoimmune diseases. Therefore, as long as the vaccination campaign advance, data have been acquired for RAEs through post-marketing surveillance systems, case reports, and observational studies—the latter allowing for a less biased risk assessment. Concerning CIDEs, a systematic review accounted for 32 events reported in the literature until September 2021 (9). Besides case descriptions, in a recent self-controlled case-series study—conducted from December 2020 to May 2021 on a cohort of more than 32 million people in England—Patone et al. (10) assessed the association between the first dose of a COVID-19 vaccine (ChAdOx1nCoV-19 or BNT162b2) and the occurrence of neurological complications, including CIDEs. Study findings showed an increased risk of Guillain–Barré syndrome and Bell's palsy after ChAdOx1nCoV-19 and of hemorrhagic stroke after BNT162b2, while CIDEs did not result associated to neither vaccine. However, a trend toward increased risk of encephalitis/meningitis/myelitis was reported after ChAdOx1nCoV-19 vaccine. Few other studies evaluated CIDEs risk more specifically in persons affected by a chronic CNS inflammatory demyelinating disease. Achiron et al. assessed the safety of BNT162b2 in an adult MS cohort (*n* = 574) in Israel, finding no increased risk of clinical relapses in a median time of 38 and 20 days, respectively, from the first and the second dose (11). Consistent results came from an Italian study (*n* = 324 patients) evaluating clinical relapse rates in a longer timeframe (2 months) after the first dose of an mRNA-based vaccine (12). Another Italian study conducted on a cohort of AQP4-positive NMOSD (*n* = 26) and MOGAD (*n* = 30) patients, showed no higher frequency of relapses in the month after an mRNA-vaccine administration (13). Although these studies did not include MRI data—detecting potential subclinical disease activity—they provide evidence supporting the COVID-19 vaccine's safety in patients affected by chronic inflammatory demyelinating diseases, encouraging their access to vaccination campaigns.

#### CIDEs characteristics

In our case series, we reported and characterized six post-COVID-19 vaccines CIDEs, including both acute syndromes (two ATM) and new diagnoses/relapses of chronic CNS inflammatory demyelinating diseases (three MS and one NMOSD). Among ATM, one case (Case 1) occurred 8 days after the first dose of Ad26.COV2.S and showed characteristics in line with the other 22 cases of ATM previously published in the literature. The other (Case 2) was instead a case of recurrent ATM presenting multiple reactivations of the same spinal cord lesion after the administration of subsequent vaccine doses. Interestingly, the patient reported his first event 3 months after receiving Tdap and IVP vaccines, while the other two reactivations occurred after BNT162b2 second and third dose, as the mechanism driving those events would be shared across different vaccine types. Notably, the patient clinically and radiologically recovered within ATM recurrences and did not present any other spinal cord/brain lesion in the timeframes between vaccine administrations. These elements would argue against a diagnosis of a chronic inflammatory demyelinating disease such as MS, but—considering the presence of CSF OCB and the recent proposal of a new pure spinal MS phenotype (71)—a longer follow-up is needed to exclude any further disease activity. Our three MS cases (Cases 3–5) occurred after the BNT162b2 vaccine. In two patients (Cases 3 and 4), the event represented the clinical onset, and diagnosis was made according to the 2017-revised McDonald criteria. Intriguingly, in Case 3, the anti-EBV antibodies serum pattern was strongly suggestive of recent primary infection/reactivation. This is particularly remarkable considering the recent findings supporting the causal role of EBV in MS (72), pointing to a possible synergic effect between EBV infection and a simultaneous/strictly sequential vaccine administration in the priming of self-reactive lymphocytes (especially with regards to vaccine adjuvant component- as discussed below). In Case 5, we described the occurrence of a relapse in a previously diagnosed MS patient, showing demographic/disease characteristics consistent with the other 31 cases of MS relapses published in the literature. Last, we reported a newly diagnosed AQP4-positive NMOSD (Case 6), in a patient—with a previous history of two optic neuritis- presenting with LETM and brainstem involvement after BNT162b2 second dose.

Considering our cases (*n* = 6) and those collected from the literature (*n* = 85), we summarized the characteristics of 91 CIDEs. Overall, age was heterogeneous (Figure 4C), especially in ATM and ADEM, in line with recent evidence showing that differently from the post-infectious variant (more frequent in childhood) ADEM following vaccinations seems to occur at any lifetime (73). Apart from ATM where no sex prevalence was observed, females represent the majority in all other CIDEs (Figure 4B), as generally expected for CNS inflammatory demyelinating diseases [except for ADEM, known to have male predominance (15)]. Concerning past medical history, 40% of ADEM and 14% of ATM cases presented a previous diagnosis of immune-mediated disease, suggesting a possible predisposition to develop a dysfunctional immune response. The majority of MS patients were clinically/radiologically stable and with mild disability at the time of vaccination. Notably, 59% of them were either not assuming treatment or on first-line DMT and therefore possibly at higher risk of disease activity compared to patients receiving high-efficacy therapies. CIDEs outcome was generally favorable in MS and ADEM (except when a hemorrhagic variant occurred), while ATM and NMOSD more likely showed partial recovery.

Overall, CIDEs were described after both mRNA-based, adenoviral-vectored, and inactivated vaccines. In ATM, adenoviral-vectored and mRNA vaccines resulted in almost equal proportions (46 vs. 42%). Contrarily, adenoviral-vectored vaccines accounted for the greatest amount of ADEM (55%) and NMOSD/MOGAD (56%), while in MS cases mRNA-vaccines resulted in the majority of both new diagnoses (87%) and relapses (56%; Figure 4A). However, results for MS relapses could be biased by the fact that mRNA-based vaccines were mainly preferred for persons with autoimmune diseases, including MS. On average, ADEM and NMOSD/MOGAD presented a longer time of onset (12.5 and 10 days) compared to ATM and MS (6 and 7 days; Figure 4D); interestingly, ATM after mRNA-based vaccines occurred earlier than those following adenoviral-vectored ones (2.5 vs. 8 days), with a similar trend observed in MS relapses (Figure 4E). Moreover, 19/26 (73%) of very early onset CIDEs (within 3 days from vaccine administration) followed an mRNA-vaccine. Although these observations are limited by small numbers and potential recording bias, both the prevalence of vaccine types for certain CIDEs and the heterogeneity in time of onset could suggest that different mechanisms might underly these events.

### Possible immunological mechanisms

Like all other vaccinations, anti-COVID-19 vaccines bear two components: a pathogen-specific antigen—against which neutralizing antibodies and specific T cells are desired, and an adjuvant— which is able to stimulate the innate immune response providing the second signal and pro-inflammatory cytokines to initiate the adaptive response. In mRNA-based vaccines, the mRNA itself constitutes both the immunogen (synthetizing the SARS-CoV-2 Spike glycoprotein) and the adjuvant (for the RNA intrinsic properties to be recognized by pattern recognition receptors (PRR), such as TLR3 and TRL7), while in adenoviral-vectored vaccines the antigen is encoded in the DNA of a recombinant Chimpanzee adenovirus and the adjuvant is provided by the virus particles itself (74). On these bases, several mechanisms—already advanced for other vaccines—could be proposed to explain post-COVID-19 vaccinations CIDEs (68, 69). For instance, vaccines, stimulating innate immune response through adjuvants and creating an inflammatory cytokines environment, could activate pre-existing self-reactive T and B cells, in a process known as bystander activation. This would occur rapidly in the early phase of the immune response and could therefore be involved in early-CIDEs presenting in the next few days after a vaccine administration, such as in early-onset ATM following mRNA vaccines. Other possible mechanisms include molecular mimicry (vaccine-derived antigens mimicking self-molecules could prime cross-reactive T cells) and epitope spreading (after the initial activation of antigen-specific T cells against a dominant epitope, the immune response could react also against different epitopes of the same or other proteins of both self and non-self origin). Theoretically, all these mechanisms would involve both a cell-mediated and a humoral adaptive immune response. Interestingly, adenoviral-vectored vaccines have been previously associated with Guillain-Barré Syndrome (75)—which is largely driven by aberrant autoantibodies (29)—and thrombotic thrombocytopenia, mediated by platelet-activating antibodies against PF4 (76); from our analysis, adenoviral-vectored formulations resulted in major amounts in NMOSD cases and all ATM with a cranial nerve palsy. Altogether, these observations could suggest a higher tendency of adenoviral-vectored vaccines to trigger antibodies-mediated diseases, compared to mRNA-based formulations. Nevertheless, considering the rarity of these events both in the general population and in persons with immune-mediated diseases—beyond the possible immunological mechanisms involved—a genetic predisposition underlying an abnormal reaction to vaccine stimuli could play a key role. In this regard, polymorphisms in TLRs and other PRRs recognizing adjuvants could potentially affect the innate immune response to immunizations and represent risk factors for RAEs, as previously suggested by some authors (77).

### CIDEs after the SARS-CoV-2 infection

Besides post-COVID-19 vaccines events, CIDEs have also been described after the SARS-CoV-2 infection itself. A systematic review reported 60 studies published from January 2020 to June 2021 describing 102 CNS demyelinating events temporally associated with COVID-19, including encephalitis/encephalomyelitis, ATM, and MS/NMOSD/MOGAD-like demyelination (78). More recently further studies reported cases of MS and NMOSD onset/relapses following SARS-CoV-2 infection (79–81), suggesting its possible ability to trigger inflammatory disease activity as previously considered for other viruses, especially with regard to MS (82, 83). In their large case-series study, Patone et al. (10) compared the risk of developing neurological complications after SARS-CoV-2 infection with that after COVID-19 vaccines, showing that the former was substantially higher. Taken together, these data further strengthen the favorable risk-benefits profile of COVID-19 vaccines, supporting their use both in the general population and in persons affected by chronic CNS inflammatory demyelinating diseases.

### Limitations

Small numbers and potential recording/reporting bias of reviewed cases hampered the feasibility of performing inferential statistics and meta-analysis, limiting our study to a descriptive level. Moreover, we could not account for the number of persons administrated with different vaccine types in the population from which cases came from. Indeed, those data were highly variable among countries/times and difficult to estimate considering the worldwide source of reviewed cases and their occurrence in different time periods. We did not summarize the long-term follow-up outcomes and possible further events following vaccine booster doses (if administered), since data were missing in most of the reports. Whether a pre-existing CIDE would represent a risk factor for a future aberrant immune response to the same/another vaccine still remains an open question—with major implications in the clinical setting.

## Conclusion

While epidemiological studies have assessed the safety of COVID-19 vaccines, detailed descriptions and systematic reviews of sporadic cases may still be valuable to gain insights into CIDEs pathophysiology and suggest candidate risk factors. From our pooled analysis, both the prevalence of vaccine types for certain CIDEs and the differences in time of onset might suggest that distinct mechanisms—with different dynamics and kinetic—could underly these events. Further large-scale observational studies are needed—both in the general population and in subgroups affected by chronic CNS inflammatory demyelinating diseases—to evaluate clinical and MRI data as well as other biomarkers (including genetic ones) potentially predicting CIDEs risk. These would help to optimize immunization strategies and tailor clinical management in patients with a history of post-vaccination CIDEs, as well as providing novel insights for future vaccine development.

## Data availability statement

The original contributions presented in the study are included in the article/Supplementary material, further inquiries can be directed to the corresponding authors.

## Author contributions

VR, GB, MS, and GR contributed to the conception and structure of the study. VR, MB, RR, MN, VZ, RN, RP, and AS contributed to clinical and MRI data collection of original cases described. VR and GB conducted the systematic literature search. VR performed pooled descriptive analysis and wrote the first draft of the manuscript. GB, VR, and AM contributed to figures and tables creation. All authors contributed to manuscript revision, read, and approved the submitted version.

## Conflict of interest

The authors declare that the research was conducted in the absence of any commercial or financial relationships that could be construed as a potential conflict of interest.

## Publisher's note

All claims expressed in this article are solely those of the authors and do not necessarily represent those of their affiliated organizations, or those of the publisher, the editors and the reviewers. Any product that may be evaluated in this article, or claim that may be made by its manufacturer, is not guaranteed or endorsed by the publisher.
