# Supplementary material for: CNS inflammatory demyelinating events after COVID-19 vaccines: A case series and systematic review
Source: Front Neurol. 2022 Dec 1;13:1018785. doi: 10.3389/fneur.2022.1018785 (PMC9752005; doi:10.3389/fneur.2022.1018785)
Supplement: Supplementary file 1 [file Table_1.DOCX]

**Supplementary table 1. Cases of ATM after COVID-19 vaccines**

|  | **Age**  **Sex** | **Past medical history** | **Vaccine  [dose]** | **Time from vaccine to**  **symptoms**  **(days)^a^** | **Clinical presentation** | **MRI^b^** | **CSF^c^** | **Serum** | **Treatment** | **Recovery**  **^d^** | **Reference**  **[study type]**  **Country** |
| --- | --- | --- | --- | --- | --- | --- | --- | --- | --- | --- | --- |
| 1 | 36  M | None | ChAdOx1 nCoV-19 (Vaxzevria)  [1] | 8 | LL and trunk paresthesia | C6-C7 Gd+ | OCB n/a  WBC -  Protein +  Infectious panel - | AQP4 - MOG - CTD - | OCS + IVMP 5 | Complete | Malhotra HS et al [CR]  India |
| 2 | 45  M | Atopic dermatitis | ChAdOx1 nCoV-19  (Vaxzevria)  [1] | 8 | Back pain, 4 limbs sensory loss and weakness, urinary retention | LETM  C3 to D2  Gd- | OCB -  WBC +++ (67% PN)  Protein +  Infectious panel –  AQP4 - MOG –  NS/ON - | AQP4 - MOG - CTD - | IVMP 5 + OCS | Almost complete | Pagenkopf C et al  [CR]  Germany |
| 3 | 44  F | None | ChAdOx1 nCoV-19  (Vaxzevria)  [1] | 4 | LL paresthesia and sensory loss | D7-D8 Gd+, D10-D11 Gd- | OCB -  WBC + (100% MN)  Protein +  Infectious panel - | AQP4 - MOG - CTD - NS/ON - | IVMP 5 + OCS | Complete | Vegezzi E et al  [CR]  Italy |
| 4 | 58  M | DM, pulmonary sarcoidosis | ChAdOx1 nCoV-19  (Vaxzevria)  [1] | 7 | LL and trunk  dysesthesia and sensory loss, LL weakness, urge incontinence | LETM  D2 to D10 Gd+ | OCB + (pattern IV)  WBC + (100% LY)  Protein +++  Cytology -  Infectious panel – | AQP4 - MOG - ANCA +^e^ NS/ON –  Infectious panel – | IVMP 5 + OCS +  PEX 5 | Partial | Notghi AA et al  [CR]  UK |
| 5 | 41  M | DM | ChAdOx1 nCoV-19  (Vaxzevria)  [1] | 21 | Bell’s palsy, LL and trunk  paresthesia and sensory loss, LL weakness | LETM  D1 to D6 Gd+ | OCB n/a  WBC + (100% LY)  Protein -  Infectious panel - | AQP4 - CTD - | IVMP 5 + OCS | Complete | Hsiao YT et al  [CR]  Taiwan |
| 6 | 25  F | None | ChAdOx1 nCoV-19  (Vaxzevria)  [1] | 12 | LL and trunk  paresthesia, LL weakness, urinary retention | D3-D5 Gd-, D7-D8 Gd+, D11-L1 Gd- | OCB -  WBC n/a  Protein +++  Infectious panel –  AQP4 –  MOG- | CTD - | IVMP 5 | Partial | Tan WJ et al  [CR]  Malaysia |
| 7 | 65  M | None | ChAdOx1 nCoV-19  (Vaxzevria)  [1] | 8 | 4 limbs weakness | C4-C6 Gd- | OCB -  WBC -  Protein +  Infectious panel –  AQP4 –  MOG- | - | IVMP 5 + OCS | Almost complete | Correa D G et al  [CS]  Brazil |
| 8 | 27  F | None | ChAdOx1 nCoV-19  (Vaxzevria)  [1] | 21 | Back pain, trunk and LL paresthesia, sensory loss, 4 limbs weakness, urinary retention | LETM  C3 to C7 Gd-;  D2-D3, D4, D7-D8, conus Gd- | OCB -  WBC +++ (98% MN)  Protein +  Infectious panel – | AQP4 - CTD – | IVMP 5 + PEX 7 | Partial | Da Silva et al  [CR]  Brazil |
| 9 | 37  F | None | ChAdOx1 nCoV-19  (Vaxzevria)  [1] | 2 | Left eye esotropia ( VI c.n. palsy), back pain, trunk and LL paresthesia, sensory loss and weakness, urinary retention | LETM  C1 to C6 Gd-, D1 to D7 Gd- | OCB n/a  WBC ++  Protein -  Infectious panel – | AQP4 –  Infectious panel – | IVMP 5 + IVIG | Partial | Kawtharani A A et al  [CR]  Lebanon |
| 10 | 44  F | None | Ad26.COV2.S (Janssen)  [1] | 10 | Back pain, LL limbs paresthesia, sensory loss and weakness, urinary retention, Bell’s palsy | LETM  C2-C3 to D1 Gd n/a | OCB -  WBC ++ (96% LY)  Protein –  Cytology -  Infectious panel – | AQP4 - CTD –  Infectious panel | IVMP 3 + PEX 5 | Complete | Tahir N et al  [CR]  USA |
| 11 | 34  M | None | Ad26.COV2.S (Janssen)  [1] | 8 | 4 limbs paresthesia, sensory loss and weakness, sphincter disturbances | LETM C3 to conus  Gd- | OCB -  WBC +++ (90% MN)  Protein +  Infectious panel – | AQP4 -  CTD - | IVMP 5 +  OCS | Partial | Rinaldi et al [this CS]  Italy |
| 12 | 69  F | Cervical cancer, Hypothyroidism, Dyslipidemia, RLS, Sciatica | BNT162b2 (Pfizer/BionTech)  [1] | 2 | UL paresthesia, 4 limbs weakness | LETM  C3-C4 to D2-D3 Gd n/a | OCB + (pattern IV)  WBC -  Protein –  Infectious panel - | CTD - NS/ON –  AQP4 –  MOG –  Cox B+^f^ | IVMP 5 | Partial | Mclean P et al  [CR]  USA |
| 13 | 26  F | Pancreatitis, Recurrent urinary tract infections | BNT162b2 (Pfizer/BionTech)  [1] | 3 | LL paresthesia and sensory loss, urinary retention | D5 Gd+ | OCB n/a  WBC + (100% LY)  Protein -  Infectious panel –  AQP4 –  MOG- | CTD –  Infectious panel – | IVMP 5 | Partial | Alabkal J et al  [CR]  Canada |
| 14 | 85  M | n/a | BNT162b2 (Pfizer/BionTech)  [2] | 3 | LL sensory loss and weakness, urinary retention | LETM D3 to D5  Gd n/a | OCB -  WBC + (MN)  Protein + Cytology -  Infectious panel – | AQP4 - | IVMP 6 + OCS | Death | Nakano et al  [CR]  Japan |
| 15 | 75  M | Hypertension, dyslipidemia, prostate cancer | BNT162b2 (Pfizer/BionTech)  [1] | 3 | Back pain, trunk and LL sensory loss and weakness, urinary retention | LETM  D10-L1 Gd+ | OCB -  WBC -  Protein +  Infectious panel – | AQP4 - MOG - CTD –  NS/ON –  Infectious panel – | IVMP 3 (2) + OCS + PEX 7 | Partial | Miyaue N et al  [CR]  Japan |
| 16 | 19  M | ATM after Tdap/IPV vaccines 3 years before with radiological reactivation after 2 dose of BNT162b2 | BNT162b2 (Pfizer/BionTech)  [3] | 35 | Right UL paresthesia, right limbs weakness | LETM  C5 to C7 Gd+ | OCB + (pattern II)  WBC –  Protein –  Infectious panel - | AQP4 - MOG - CTD –  NS/ON –  Infectious panel – | IVMP 5 + OCS | Complete | Rinaldi V et al [this CS]  Italy |
| 17 | 67  M | CAD, CKD, neuropathy, colon rupture | mRNA-1273 (Moderna)  [1] | 1 | LL paresthesia and sensory loss, 4 limbs weakness | LETM  C1 to C3  Gd+ | OCB + (pattern IV)  WBC -  Protein +  Infectious panel –  AQP4 –  NS/ON – | AQP4 - MOG - CTD –  Infectious panel – | IVMP 3 + PEX 5 | Partial | Khan E et al  [CR]  India |
| 18 | 76  F | AH, hearing loss | mRNA-1273 (Moderna)  [1] | 2 | Right limbs paresthesia, LL sensory loss | LETM  C2 to C5  Gd+ | OCB -  WBC + (73% PM)  Protein + Cytology -  Infectious panel – | AQP4 - CTD – | IVMP 5 + OCS | Complete | Gao JJ et al  [CR]  Taiwan |
| 19 | 70  M | AH, Hyperuricemia, Alcoholic liver cirrhosis | mRNA-1273 (Moderna)  [1] | 7 | LL and trunk sensory loss, LL weakness | D1-D2 Gd+, D5-D6 Gd- | OCB + (pattern II)  WBC -  Protein +  Infectious panel –  AQP4 - NS/ON – | AQP4 - MOG - CTD -  Infectious panel – | IVMP 5 + OCS | Complete | Hirose S et al  [CR]  Japan |
| 20 | 67  F | n/a | mRNA-1273 (Moderna)  [1] | 1 | LL weakness | LETM  C1 to C3 Gd+ | OCB -  WBC -  Protein - | AQP4 - MOG - | IVIG +  PEX | Partial | Sriwastava S et al  [CS]  USA |
| 21 | 46  F | B12 deficiency in treatment | mRNA-1273 (Moderna)  [1] | 2 | Back pain, trunk and LL paresthesia and sensory loss, 4 limbs weakness, urinary retention | LETM  C6 to D2 | OCB n/a  WBC -  Protein -  Infectious panel – | AQP4 - CTD - | IVMP + OCS | Partial | Fujikawa P et al  [CR]  USA |
| 22 | 78  F | AH, DM, breast cancer | CoronaVac (Sinovac)  [2] | 21 | UL paresthesia, 4 limbs weakness, urinary retention | LETM  C1 to D3 Gd n/a | OCB -  WBC -  Protein + | AQP4 - MOG - CTD -  Infectious panel – | IVMP 4 + PEX | Partial | Erdem S N et al  [CR]  Turkey |
| 23 | 61  F | AH, asthma | CoronaVac (Sinovac)  [2] | 5 | 4 limbs sensory loss and weakness | C3-C5 Gd+ | OCB n/a  WBC -  Protein - | AQP4 - CTD -  Infectious panel – | IVMP 5 +  OCS | Partial | Khan Z et al  [CR]  Pakistan |
| 24 | 71  M | AH, DM, IHD | BBIBP-CorV (Sinopharm)  [1] | 5 | Right limbs sensory loss, left limbs weakness, urinary retention | LETM  C1 to C3 Gd- | OCB n/a  WBC -  Protein - | AQP4 - MOG –  CTD -  Infectious panel – | IVMP 6 | Almost complete | Sepahvand M et al [CR]  Iran |

AH, arterial hypertension; AQP4, anti-aquaporin-4 antibodies; CAD, coronary artery disease; CKD, chrnoic kidney disease; c.n. cranial nerve; CR, case report; CS, case series ; CSF, cerebrospinal fluid; CTD, connective tissue disease; DM, diabetes mellitus; IHD, ischemich heart disease; IPV, inactivated polio vaccine; IVIG, intravenous immunoglobulin; IVMP, high dose intravenous methyilprednisolone; LL, lower limbs; LY, lymphocytes; MN, mononuclear cells; MOG, anti-myelin oligodendrocyte glycoprotein antibodies; n/a, data not available; NS/ON, anti-neuronal surface/onconeural antibodies; OCB, oligoclonal bands; OCS, oral corticosteroid; PEX, plasma exchange; PN, polymorphonuclear cells; RLS, restless legs syndrome; Tdap, adult dose of tetanus, diphteria, pertussis vaccine; UL, upper limbs.

^a^ Timeframe between vaccine administration and onset of ATM symptoms
^b^ Lesions localization, extension (LETM, longitudinally extending transverse myelitis) and gadolinium enhancement (Gd+/Gd-)
^c^ CSF WBC and protein levels were expressed with –/+/++/+++ for normal or mildly/moderately/markedly elevated levels, considering as value ranges 0-5, 6-25, 26-100, >100 /μL for WBC and 0-45, 46-150, 151-300, >300 mg/dl for protein levels

^d^ Recovery at last available follow-up
^e^ Notghi et al reported atypical ANCA positivity of doubtful significance with negative anti-PR3 and MPO antibodies
^f^ McLean et al reported Coxsackie B5 positivity (titers of 1:8) and Coxsackie B6 (titers of 1:16) that was not considered significant since the low titers and the absence of clinical history suggestive of Coxsackie infection (rash or viral pro- drome).
