# Supplementary material for: CNS inflammatory demyelinating events after COVID-19 vaccines: A case series and systematic review
Source: Front Neurol. 2022 Dec 1;13:1018785. doi: 10.3389/fneur.2022.1018785 (PMC9752005; doi:10.3389/fneur.2022.1018785)
Supplement: Supplementary file 2 [file Table_2.DOCX]

**Supplementary table 2. Cases of ADEM after COVID-19 vaccines**

|  | **Age**  **Sex** | **Past medical history** | **Vaccine  [dose]** | **Time from vaccine to**  **symptoms**  **(days)^a^** | **Clinical presentation** | **MRI^b^** | **CSF^c^** | **Serum** | **Treatment** | **Recovery^d^** | **Reference**  **[study type]**  **Country** |
| --- | --- | --- | --- | --- | --- | --- | --- | --- | --- | --- | --- |
| 1 | 45  M | None | ChAdOx1 nCoV-19 (Vaxzevria)  [1] | 12 | 4 limbs and trunk paresthesia, right UL weakness, bilateral blurred vision, dysarthria, dysphagia, urge incontinence | 1 large IT (pons and MCP) Gd+; 1 thalamic Gd-; >3 SC Gd +, 1 SC Gd - | OCB + (pattern II)  WBC ++ (98%MN)  Protein -  Infectious panel - | AQP4 - MOG - CTD - NS/ON – | IVMP 5 + OCS | Complete | Rinaldi V et al  [CR]  Italy |
| 2 | 63  M | DM, ischemic heart disease, atrial fibrillation | ChAdOx1 nCoV-19 (Vaxzevria)  [1] | 12 | Vertigo, abdominal pain, fatigue, decreased level of consciousness | >20 ST Gd n/a | - | - | IVMP + PEX | Death | Permezel F et al  [CR]  Australia |
| 3 | 61  M | Hypothyroidism, polymyalgia rheumatica | ChAdOx1 nCoV-19 (Vaxzevria)  [1] | 2 | Fever, headache, apathy, seizure | Multiple large ST (cortical, DWM) Gd n/a; thalamic hemorrhagic focus | OCB -  WBC -  Protein n/a Infectious panel - | AQP4 - MOG - CTD - NS/ON – | IVMP 5 + PEX 7 + OCS | Poor | Ancau M et al  [CS]  Germany |
| 4 | 25  F | None | ChAdOx1 nCoV-19 (Vaxzevria)  [1] | 9 | Headache, back pain, LL paresthesia, sensory loss and weakness, urinary retention | Multiple ST Gd+; thoracic LETM with hemorrhagic focus, Gd+ | OCB -  WBC +++ (PN)  Protein n/a Infectious panel - | AQP4 - MOG - CTD - NS/ON – | IVMP 5 + PEX 7 + OCS | Partial | Ancau M et al  [CS]  Germany |
| 5 | 55  F | n/a | ChAdOx1 nCoV-19 (Vaxzevria)  [1] | 9 | Nausea, dizziness, 4 limbs weakness, coma | Multiple ST with hemorrhagic foci, Gd n/a | OCB -  WBC + (PN)  Protein n/a  Infectious panel - | AQP4 - MOG - NS/ON – | IVMP 5 (2) | Death | Ancau M et al  [CS]  Germany |
| 6 | 29  F | None | Gam-COVID-Vac (Sputnik V)  [2] | 7 | Fever, vomiting, memory loss, behavioral disturbances, right limbs weakness | Multiple large ST (DWM/iuxtacortical) Gd+ | OCB -  WBC -  Protein -  Infectious panel - | AQP4 - MOG -  CTD –  Infectious panle - | IVMP 5 + PEX 7 + OCS | Partial | Shalilahmdi D et al  [CR]  Iran |
| 7 | 88  F | DM, Alzheimer disease | BNT162b2 (Pfizer/BionTech)  [2] | 29 | Decreased level of consciousness, nystagmus | 2 IT (bilateral MCP), Gd+ | OCB -  WBC n/a  Protein n/a  Infectious panel - | CTD - NS/ON –  AG - | IVMP 3 | Complete | Shimizu M et al  [CR]  Japan |
| 8 | 56  F | Post-infectious rhombencephalitis 5 years before, Herpes Zoster | BNT162b2 (Pfizer/BionTech)  [1] | 13 | Malaise, left UL weakness and left limbs ataxia | 1 IT (MCP) Gd-; >2 ST Gd- | OCB -  WBC -  Protein -  NS/ON –  Cytokine panel: CSF/serum >1 for IL6, IL10, IFNγ | AQP4 –  MOG –  CTD - NS/ON –  AG -  Infectious panel – | OCS | Almost complete | Vogrig A et al  [CR]  Italy |
| 9 | 19  F | Atopic dermatitis, depression | mRNA-1273 (Moderna)  [1] | 14 | Fever, headache, nausea/vomiting, back/neck pain, urinary retention | Multiple large ST (DWM, cortical) and IT Gd-, >3 large ST Gd+; LETM (C1 to D11) Gd+ | OCB -  WBC +++ (91% LY)  Protein +  Infectious panel – | AQP4 –  MOG- | IVMP | Almost complete | Kania K et al  [CR]  Poland |
| 10 | 24  F | None | BBIBP-CorV (Sinopharm)  [1] | 14 | Fever, headache, muscle stiffness, extremities weakness, lethargy, memory loss, seizure | Multiple ST (cortical) Gd- | OCB -  WBC ++  Protein n/a  Infectious panel –  AQP4 - MOG - NS/ON – | CTD –  Infectious panel – | IVIG 5 | Complete | Cao L et al  [CR]  China |
| 11 | 46  F | Hashimoto’s thyroiditis | BBIBP-CorV (Sinopharm)  [2] | 30 | Seizure, pyramidal signs | Multiple large ST (DWM, cortical, thalamic) Gd- | OCB -  WBC -  Protein -  Infectious panel – | AQP4 –  MOG -  CTD: ANA 1/100, SOX1 + | IVMP 7 + OCS | Complete | Kenangil GO et al  [CR]  Turkey |

DM, diabetes mellitus; IVIG, intravenous immunoglobulin; IVMP, high dose intravenous methylprednisolone; LL, lower limbs; LY, lymphocytes; MN, mononuclear cells; MOG, anti-myelin oligodendrocyte glycoprotein antibodies; n/a, data not available; NS/ON, anti-neuronal surface/onconeural antibodies; OCB, oligoclonal bands; OCS, oral corticosteroid; PEX, plasma exchange; PN, polymorphonuclear cells; UL, upper limbs.

^a^ Timeframe between vaccine administration and onset of ADEM symptoms (including typical prodromal symptoms as fever, headache, malaise, nausea and vertigo)
^b^ Lesions number, localization (ST, supratentorial; IT, infratentorial; DWM, deep white matter; MCP, middle cerebellar peduncle; SC, spinal cord; LETM, longitudinally extending transverse myelitis), presence of hemorrhagic foci and enhancement to gadolinium (Gd+/Gd-)
^c^ CSF WBC and protein levels were expressed with –/+/++/+++ for normal or mildly/moderately/markedly elevated levels, considering as value ranges 0-5, 6-25, 26-100, >100 /μL for WBC and 0-45, 46-150, 151-300, >300 mg/dl for protein levels

^d^ Recovery at last available follow-up
