# Supplementary material for: CNS inflammatory demyelinating events after COVID-19 vaccines: A case series and systematic review
Source: Front Neurol. 2022 Dec 1;13:1018785. doi: 10.3389/fneur.2022.1018785 (PMC9752005; doi:10.3389/fneur.2022.1018785)
Supplement: Supplementary file 3 [file Table_3.DOCX]

**Supplementary table 3. Cases of MS after COVID-19 vaccines**

|  | **Age**  **Sex** | **Past medical history** | **Disease pheno-**  **type** | **Disease**  **duration** | **Time since last relapse**  **(days)^a^** | **EDSS**  **^b^** | **DMT** | **Vaccine**  **[dose]** | **Time from vaccine to**  **symptoms^c^** | **Clinical presenta**  **tion** | **MRI^d^** | **CSF^e^** | **Serum** | **Treat**  **ment** | **Reco**  **very^f^** | **Reference (study type)**  **Country** |
| --- | --- | --- | --- | --- | --- | --- | --- | --- | --- | --- | --- | --- | --- | --- | --- | --- |
| 1 | 33  M | None | Newly  diagno  sed MS | - | - | n/a | None | BNT162b2 (Pfizer/BionTech)  [2] | 1 day | Left eye blurred vision | >7 ST (PV, IC) Gd-;  1 ST Gd+; 1 SC Gd- | OCB +  WBC -  Protein - | AQP4 - | IVMP  3  + OCS | Complete | Khayat‐Khoei M et al  [CS]  USA |
| 2 | 28  F | None | Newly  diagno  sed MS | - | - | n/a | None | BNT162b2 (Pfizer/BionTech)  [1] | 6 days | Trunk and LL sensory loss, left LL weakness | >20 brain Gd-; 1 SC Gd+ | OCB +  WBC - | - | IVMP 10  + PEX | Partial | Havla J et al  [CR]  Germany |
| 3 | 45  F | Hypothyroidism | Newly  diagno  sed MS | - | - | n/a | None | BNT162b2 (Pfizer/BionTech)  [1] | 7 days | LL paresthesia, left LL weakness, gait ataxia | Multiple PV Gd n/a | OCB + | - | IVMP  5  + OCS | Complete | Watad A et al  [CS]  Israel |
| 4 | 32  F | None | Newly  diagno  sed MS | - | - | n/a | None | BNT162b2 (Pfizer/BionTech)  [1] | 3 days | Right limbs sensory loss and weakness, dysarthria, gait ataxia | Multiple ST (PV), 1 IT Gd n/a | OCB + | WBC 11.300/μL  CRP - | IVMP  3  + OCS | Complete | Tagliaferri AR et al  [CS]  USA |
| 5 | 40   F | Facial palsy 4 years before | Newly  diagno  sed MS | - | - | n/a | None | BNT162b2 (Pfizer/BionTech)  [2] | 14 days | Right UL paresthesia | Multiple ST (PV) Gd-; 1 SC Gd+ | OCB +  WBC -  Protein – | AQP4–  MOG –  CTD - | IVMP  3 | Complete | Fujimori J et al  [CR]  Japan |
| 6 | 29  F | Migraines | Newly  diagno  sed MS | - | - | n/a | None | BNT162b2 (Pfizer/BionTech)  [1] | 1 day | Left LL weakness, left LL and right UL paresthesia | Multiple ST (PV, IC) Gd-; 1 ST Gd+ | OCB +  WBC + | CTD - | IVMP  5 | Signifi  cant improvement | Toljan K et al  [CS]  USA |
| 7 | 37   M | None | Newly  diagno  sed MS | - | - | n/a | None | BNT162b2 (Pfizer/BionTech)  [1] | 3 days | Left UP paresthesia, urinary urgency, gait ataxia | Multiple PV n/a; 1 SC n/a | - | AQP4 –  MOG - | IVMP  5  + OCS | n/a | Toljan K et al  [CS]  USA |
| 8 | 43  F | n/a | Newly  diagno  sed MS | - | - | n/a | None | BNT162b2 (Pfizer/BionTech)  [2] | 35 days | Right UL weakness, right face paresthesia | Multiple ST (PV) Gd+ and Gd-; V c.n. Gd+ | OCB + WBC - | CTD - | IVMP 3 | Almost complete | Toljan K et al  [CS]  USA |
| 9 | 40  F | Renal cell carcino  ma | Newly  diagno  sed MS | - | - | n/a | None | BNT162b2 (Pfizer/BionTech)  [1] | 10 days | UP paresthe  sia, diplopia | 1 IT Gd+, 1 SC Gd- | OCB +  WBC -  Protein –  Infectious panel - | AQP4–  MOG –  CTD - | IVMP 5 | Almost complete | Rinaldi V et al  [this CS] |
| 10 | 27  F | None | Newly  diagno  sed MS | - | - | n/a | None | BNT162b2 (Pfizer/BionTech)  [2] | 7 days | Left LL, right face/ limbs weakness, dysarthria, Lhermitte’s | 2 ST (PV) Gd+; 2 ST Gd -; 3 SC Gd- | OCB +  WBC -  Protein –  Infectious panel - | AQP4–  CTD: ANA + (1:160) | IVMP 5 (2) + OCS + PEX 5 + IVMP 3 | Almost complete | Rinaldi V et al  [this CS]  Italy |
| 11 | 26  F | None | Newly  diagno  sed MS | - | - | n/a | None | mRNA-1273 (Moderna)  [2] | 14 days | Right eye pain/blurred vision | 8 brain (PV, IT) Gd-; 1 ST Gd+;  1 SC Gd-, 1 SC Gd+ | OCB –  IgG index +  WBC + (LY 91%)  Protein - | CTD - | IVMP 5 | Complete | Khayat‐Khoei M et al  [CS]  USA |
| 12 | 41  M | None | Newly  diagno  sed MS | - | - | n/a | None | mRNA-1273 (Moderna)  [2] | 30 days | LL paresthesia, LL and right face/UL weakness, urinary retention | Multiple ST (PV, IC) Gd+ and Gd-; multiple SC Gd+ and Gd- | OCB +  WBC +++  Protein – | AQP4–  MOG –  CTD –  Infectious panel - | IVMP 5  + PEX 5 | Partial | Toljan K et al  [CS]  USA |
| 13 | 46  F | Optic neuritis 8 years before | Newly  diagno  sed MS | - | - | n/a | None | mRNA-1273 (Moderna)  [2] | 3 days | Trunk and UL dysesthesia | Multiple ST (PV, IC) Gd-; 1 PV Gd+; multiple SC Gd+ | OCB +  WBC +  Infectious panel - | AQP4–  MOG –  CTD – | IVMP 5 | n/a | Toljan K et al  [CS]  USA |
| 14 | 48  F | n/a | Newly  diagno  sed MS | - | - | n/a | None | ChAdOx1 nCoV-19 (Vaxzevria)  [1] | 8 days | Right eye blurred vision | Multiple ST (PV) Gd-, 1 PV Gd+ | - | - | IVMP 5 | Almost complete | Nistri R et al  [CS]  Italy |
| 15 | 66  F | n/a | Newly  diagno  sed MS | - | - | n/a | None | ChAdOx1 nCoV-19 (Vaxzevria)  [1] | 3 days | Blurred vision, gait ataxia | Multiple ST (PV) Gd-, 4 ST (PV) Gd+ | OCB + | - | IVMP 5 | Partial | Nistri R et al  [CS]  Italy |
| 16 | 48  F | n/a | CIS | 8 years | 8 years (c) | 1 | None | BNT162b2 (Pfizer/BionTech)  [1] | 15 days | Right eye pain, gait ataxia, Lhermitte’s | 3 ST Gd- | - | - | OCS | Almost complete | Khayat‐Khoei M et al  [CS]  USA |
| 17 | 24  F | n/a | RRMS | 9 years | 2.2 years (c) | 0 | Fingo  limod | BNT162b2 (Pfizer/BionTech)  [2] | 1 day | Right eye pain/blurred vision | 8 ST Gd-; 2 ST Gd+ | - | - | IVMP 3 | Complete | Khayat‐Khoei M et al  [CS]  USA |
| 18 | 31  F | n/a | dMS | 5 years | 2.3 years (c/r) | n/a | Cladribina | BNT162b2 (Pfizer/BionTech)  [1] | 2 days | Left UL paresthesia left limbs sensory loss and weakness | 3 ST Gd+ | - | - | IVMP 5 | Complete | Maniscalco GT et al  [CR]  Italy |
| 19 | 49  F | n/a | dMS | 8 years | 8 years  (c/r) | 1.5 | DMF | BNT162b2 (Pfizer/BionTech)  [1] | 5 days | Left face and UL paresthesia | 1 ST Gd+; 1 SC Gd+ | - | - | IVMP 5 | Almost complete | Nistri R et al  [CS]  Italy |
| 20 | 39  M | n/a | dMS | 7 years | 3 years (c/r) | 2 | DMF | BNT162b2 (Pfizer/BionTech)  [1] | 10 days | Left LL paresthesia | 2 ST Gd+; 1 SC Gd- | - | - | OCS | Partial | Nistri R et al  [CS] |
| 21 | 39  F | n/a | CIS | 3 years | 3 years (c/r) | 1 | None | BNT162b2 (Pfizer/BionTech)  [1] | 3 days | Right limbs dysesthesia | 1 IT Gd+ | - | - | IVMP 5 | Complete | Nistri R et al  [CS]  Italy |
| 22 | 60  F | n/a | dMS | 23 years | 6 years  (c/r) | 3.5 | DMF | BNT162b2 (Pfizer/BionTech)  [1] | 2 days | LL paresthesia fatigue | 1 ST Gd+ | - | - | None | n/a | Nistri R et al  [CS] |
| 23 | 30  F | n/a | dMS | 3 years | 1 year  (c/r) | 1.5 | Cladribina | BNT162b2 (Pfizer/BionTech)  [2] | 20 days | Aphasia | 2 ST Gd+ (1 tumefactive) | - | - | IVMP 5 | Complete | Nistri et al  [CS]  Italy |
| 24 | 58  F | n/a | dMS | 21 years | 3 years (c/r) | 5 | None | BNT162b2 (Pfizer/BionTech)  [1] | 3 days | Gait ataxia, urge incontinence, dysphagia | 1 ST Gd+ | - | - | IVMP 5 | Complete | Nistri R et al  [CS] |
| 25 | 34  F | n/a | dMS | 3 months | 3 months  (c) | 2.5 | None | BNT162b2 (Pfizer/BionTech)  [2] | 4 days | Neck pain,  Right UL sensory loss | 3 ST Gd+; 1 SC Gd- | - | - | CS | n/a | Nistri R et al  [CS]  Italy |
| 26 | 35  F | n/a | dMS | 16 years | 2 years  (c/r) | 2 | DMF | BNT162b2 (Pfizer/BionTech)  [2] | 1 day | Left limbs paresthesia | 3 ST Gd+ | - | - | CS | n/a | Nistri R et al  [CS] |
| 27 | 54  M | n/a | dMS | 18 years | 1 year  (c/r) | 2 | Teriflunomide | BNT162b2 (Pfizer/BionTech)  [1] | 7 days | Right limbs weakness | 2 ST Gd+ | - | - | IVMP 5 | Complete | Nistri R et al  [CS]  Italy |
| 28 | 37  M | n/a | dMS | 2 years | 2 years  (c) | 1.5 | DMF | BNT162b2 (Pfizer/BionTech)  [2] | 10 days | Right limbs weakness | 1 ST Gd+ | - | - | IVMP 5 | Partial | Nistri R et al  [CS]  Italy |
| 29 | 53  F | None | dMS | 21 years | 7 years  (c/r) | 2.5 | Fingolimod | BNT162b2 (Pfizer/BionTech)  [1] | 14 days | Gait ataxia, fatigue | 1 ST Gd+ | - | - | IVMP 5 | Complete | Rinaldi V et al  [this CS]  Italy |
| 30 | 35  F | n/a | RRMS | 11 years | 7 years (c/r) | 1 | Natalizu  mab | mRNA-1273 (Moderna)  [2] | 21 days | Right UL and gait ataxia | 1 IT Gd+ | - | JCV –  ANTZ - | IVMP 5 | Complete | Khayat‐Khoei M et al  [CS]  USA |
| 31 | 44  F | n/a | RRMS | 24 years | 14 years (c/r) | 1 | None | mRNA-1273 (Moderna)  [2] | 6 days | LL paresthesia right limbs weakness | 1 ST Gd+ | - | - | IVMP 3 | Complete | Khayat‐Khoei M et al  [CS]  USA |
| 32 | 42  F | n/a | dMS | 2 years | 2 years  (c/r) | 4 | Ocreli  zumab | mRNA-1273 (Moderna)  [1] | 14 days | Left UL weakness | 1 ST Gd+ | - | - | None | n/a | Nistri R et al  [CS]  Italy |
| 33 | 57  M | n/a | dMS | 20 years | 6 years  (c/r) | 6 | None | mRNA-1273 (Moderna)  [2] | 14 days | LL weakness | 1 IT Gd+ | - | - | IVMP 5 | Partial | Nistri R et al  [CS]  Italy |
| 34 | 40  F | n/a | RRMS | 21 years | 12 years  (c) | n/a | Natalizu  mab | ChAdOx1 nCoV-19 (Vaxzevria)  [1] | 14 days | Bilateral vision loss, LL weakness, trunk and LL sensory loss, urge incontinence | Bilateral ON/chiasm/tract Gd-, LETM Gd + | WBC +++ (PM)  Protein ++  AQP4 - | AQP4 - MOG –  CTD –  Infectious panel - | IVMP+ PEX | Partial | Helmchen et al  [CR]  Germany |
| 35 | 24  F | n/a | CIS | 4 years | 4 years  (c) | n/a | None | ChAdOx1 nCoV-19 (Vaxzevria)  [2] | 5 days | Left limbs paresthesiaLhermitte’s sign | >1 ST, 1 Gd+;  >1 SC, 1 Gd+ | - | - | IVMP 5  + OCS | Almost complete | Mathew T et al  [CR]  India |
| 36 | 22  F | n/a | RRMS | n/a | 5 years  (c/r) | n/a | Fingo  limod | ChAdOx1 nCoV-19 (Vaxzevria)  [1] | 7 days | Facial palsy, limbs weakness, gait ataxia | 1 tumefactive IT Gd- | - | - | IVMP | Partial | Fragoso YD et al  [CS]  Brazil |
| 37 | 32  F | n/a | RRMS | n/a | 2 years (c/r) | n/a | DMF | ChAdOx1 nCoV-19 (Vaxzevria)  [1] | 10 days | Left eye vision loss | 1 ON Gd+ | - | - | IVMP | Partial | Fragoso YD et al  [CS]  Brazil |
| 38 | 35  M | n/a | SPMS | n/a | 3 years (c/r) | n/a | Nata  lizu  mab | ChAdOx1 nCoV-19 (Vaxzevria)  [1] | 7 days | LL weakness | New lesions Gd n/a | - | - | OCS | Partial | Fragoso YD et al  [CS] |
| 39 | 30  F | n/a | RRMS | n/a | 1 year (c/r) | n/a | Nata  lizu  mab | ChAdOx1 nCoV-19 (Vaxzevria)  [1] | 25 days | Right limbs weakness | New lesions Gd+ | - | - | IVMP | Complete | Fragoso YD et al  [CS]  Brazil |
| 40 | 42  F | n/a | RRMS | n/a | 3 years (c/r) | n/a | Fingo  limod | ChAdOx1 nCoV-19 (Vaxzevria)  [1] | 15 days | UL weakness | 1 SC Gd+ | - | - | IVMP | Complete | Fragoso YD et al  [CS]  Brazil |
| 41 | 35  M | n/a | RRMS | n/a | 4 years (c/r) | n/a | Teriflunomide | ChAdOx1 nCoV-19 (Vaxzevria)  [1] | 20 days | Right UL ataxia | 1 IT Gd+ | - | - | IVMP | Partial | Fragoso YD et al  [CS]  Brazil |
| 42 | 51  M | n/a | PPMS | n/a | 2 years  (c/r) | n/a | None | ChAdOx1 nCoV-19 (Vaxzevria)  [1] | 25 days | UL sensory loss | >1 SC Gd+ | - | - | None | Partial | Fragoso YD et al  [CS]  Brazil |
| 43 | 32  F | n/a | RRMS | n/a | 6 years (c/r) | n/a | GA | ChAdOx1 nCoV-19 (Vaxzevria)  [1] | 7 days | Right limbs sensory loss and weakness | New lesions Gd+ | - | - | IVMP | Partial | Fragoso YD et al  [CS]  Brazil |
| 44 | 45  M | n/a | dMS | 9 years | 1 year  (c/r) | 2.5 | Ocreli  zumab | ChAdOx1 nCoV-19 (Vaxzevria)  [1] | 21 days | LL disesthesia | 2 ST Gd-; 1 SC Gd+ | - | - | OCS | n/a | Nistri R et al  [CS]  Italy |
| 45 | 54  F | n/a | dMS | 28 years | 7 years  (c/r) | 2.5 | None | ChAdOx1 nCoV-19 (Vaxzevria)  [1] | 3 days | Trunk and LL sensory loss | 1 SC Gd+ | - | - | IVMP 5 | Complete | Nistri R et al  [CS]  Italy |
| 46 | 34  F | n/a | dMS | 13 years | 2 years  (c) | n/a | Rituximab | Gam-COVID-Vac (Sputnik V)  [1] | 3 days | Right limbs weakness, gait ataxia | Multiple ST/IT Gd- | - | - | IVMP 5  + OCS | Complete | Etemadifar M et al  [CR]  Iran |
| 47 | 42  F | n/a | RRMS | 20 years | 6 years  (c) | n/a | None | BBIBP-CorV (Sinopharm)  [1] | 2 days | LL weakness, gait ataxia | Multiple ST/IT Gd-; 1 IT Gd+ | - | - | IVMP 5 | n/a | Seyed Ahadi MS et al  [CR]  Iran |

ANTZ, anti-Natalizumab antibodies; AQP4, anti-aquaporin-4 antibodies; CSF, cerebrospinal fluid; CTD, connective tissue disease; DMF, dimethyil fumarate; DMT, disease modifying therapy; GA, glatiramer acetate; IVMP, intravenous methyilprednisolone; JCV, anti-JCV antibodies; LL, lower limbs; LY lymphocytes; MN mononuclear cells; MOG, anti-myelin oligodendrocyte glycoprotein antibodies; n/a, data not available; OCB, oligoclonal bands; OCS oral corticosteroid; PEX, plasma exchange; PN, polymorphonuclear cells; RRMS, relapsing-remitting MS; UL, upper limbs.

^a^ Timeframe since last clinical and/or radiological relapse. (c): authors considered just clinical relapses, (c/r): both clinical and radiological relapses

^b^ Expanded Disability Status Scale at baseline
^c^ Timeframe between vaccine administration and onset of MS symptoms

^d^ New lesions number, localization (ST, supratentorial; PV, periventricular; C/I, cortical/iuxtacortical; IT, infratentorial; SC, spinal cord; LETM, longitudinally extending transverse myelitis; ON, optic nerve) and to gadolinium enhancement (Gd+/Gd-)
^e^ CSF WBC and protein levels were expressed with –/+/++/+++ for normal or mildly/moderately/markedly elevated levels, considering as value ranges 0-5, 6-25, 26-100, >100 /μL for WBC and 0-45, 46-150, 151-300, >300 mg/dl for protein levels
^f^ Recovery at last available follow-up
