# Supplementary material for: CNS inflammatory demyelinating events after COVID-19 vaccines: A case series and systematic review
Source: Front Neurol. 2022 Dec 1;13:1018785. doi: 10.3389/fneur.2022.1018785 (PMC9752005; doi:10.3389/fneur.2022.1018785)
Supplement: Supplementary file 4 [file Table_4.DOCX]

**Supplementary table 4. Cases of NMOSD and MOGAD after COVID-19 vaccines**

|  | **Age**  **Sex** | **Past medical history/**  **comorbidities** | **Disease pheno**  **type** | **Disease duration** | **Time since last relapse** | **DMT** | **Vaccine  [dose]** | **Time from vaccine to**  **symptoms(days)**  **^a^** | **Clinical presentation** | **MRI^b^** | **CSF^c^** | **Serum** | **Treatment** | **Recovery^d^** | **Reference**  **[study type]**  **Country** |
| --- | --- | --- | --- | --- | --- | --- | --- | --- | --- | --- | --- | --- | --- | --- | --- |
| 1 | 46  F | None | Newly diagnosed  NMOSD | - | - | - | ChAdOx1 nCoV-19  (Vaxzevria)  [1] | 10 | Right LL sensory loss and weakness | C2-C3 Gd+; 1 medulla, 1 lateral ventricle peri-ependymal Gd+ | OCB –  WBC -  Protein -  Infectious panel n/a | AQP4 + | IVMP + OCS | Partial | Anamnart C et al  [CS]  Thailand |
| 2 | 47  F | None | Newly diagnosed  NMOSD | - | - | - | ChAdOx1 nCoV-19  (Vaxzevria)  [1] | 10 | Trunk and 4 limbs paresthesia and sensory loss, 4 limbs weakness, urinary retention, dysarthria, disphagia | LEMT (C1-D5) Gd+; 1 right MCP/IV ventricle periependymal Gd+; 1 ST Gd- | OCB –  WBC +++ (80% PN)  Protein -  Infectious panel n/a | AQP4 - MOG - CTD - Infectious panel - | IVMP +  OCS + PEX + IVIG | Partial | Gorgone G et al  [CR]  Italy |
| 3 | 34  M | None | Newly diagnosed  NMOSD | - | - | - | Gam-COVID-Vac (Sputnik V)  [2] | 21 | Dizziness, gait ataxia, altered mental status | 1 IV and III ventricles peri-ependymal, 1 left thalamus, 1 corpus callosum, 1 optic chiasma | OCB –  WBC + (LY)  Protein +  Infectious panel - | AQP4 +  CTD –  Infectious panel - | PEX 5 | Gradually improved | Badrawi N et al  [CR]  United Arabs Emirates |
| 4 | 64  M | None | Newly diagnosed  NMOSD | - | - | - | BNT162b2 (Pfizer/BionTech)  [1] | 18 | Trunk and LL pain paresthesia, gait ataxia, urinary retention, constipation | 6 brain Gd-; LETM (cervical to conus) Gd+ | AQP4 +  OCB –  WBC -  Protein -  Infectious panel n/a | AQP4 + MOG - CTD: SSA/B+^a^ | IVMP 3 + PEX 5 | Partial | Khayat‐Khoei M et al  [CS]  USA |
| 5 | 75  F | Right ON 15 years before, bilateral ON 10 years before, AH, DM, osteoporosis | Newly diagnosed  NMOSD | - | - | OCS | BNT162b2 (Pfizer/BionTech)  [2] | 10 | 4 limbs weakness, right UL dysesthesia, LL sensory loss | Confluent PV Gd-; 1 IT Gd+; 1 LETM (C2 to C7) Gd+ | OCB + (pattern II)  WBC –  Protein –  Infectious panel –  NS/ONA - | AQP4 + MOG - CTD –  NS/ONA - | IVMP 5 | Partial | Rinaldi et al  [this CS]  Italy |
| 6 | 26  F | None | Newly diagnosed  NMOSD | - | - | - | CoronaVac (Sinovac)  [1] | 10 | Left limbs paresthesia, sensory loss and weakness | C4-C5 Gd+ | OCB –  WBC -  Protein -  Infectious panel n/a | AQP4 + | IVMP + PEX + OCS | Partial | Anamnart C et al  [CS]  Thailand |
| 7 | Middle-aged  F | None | Newly diagnosed  NMOSD | - | - | - | Inactivated  [1] | 3 | Vomiting, dizziness, gait ataxia | 1 area postrema, bilateral hypothalamus Gd- | OCB –  WBC ++ (MN)  Protein -  Infectious panel n/a | AQP4 + MOG - CTD: ANA+, SSA/B+, pANCA+, Ro-52+  NS/ONA –  Cytokine panel - | IVMP 5 | Almost complete | Chen S et al  [CR]  China |
| 8 | 62  F | None | dNMOSD | 8 | n/a | Azathioprine | ChAdOx1 nCoV-19  (Vaxzevria)  [1] | 7 | Left eye loss of vision | 1 ON Gd+ | - | - | IVMP | Complete | Fragoso YD et al  [CS]  Brazil |
| 9 | 59  M | None | Newly diagnosed  MOGAD | - | - | - | ChAdOx1 nCoV-19  (Vaxzevria)  [1] | 13 | LL paresthesia and sensory loss, gait ataxia, urinary retention | LETM (cervical, D7 to L1, conus) Gd- | OCB + (pattern IV)  WBC +++ (LY)  Protein + | AQP4 - MOG + | IVMP 5 +  PEX 5 | Partial | Dams L et al  [CR]  Germany |

AH, arterial hypertension; AQP4, anti-aquaporin-4 antibodies; CR, case report; CS, case series; CSF, cerebrospinal fluid; CTD, connective tissue disease; DM, diabetes melllitus; dNMOSD, definite neuromyelitis optica spectrum disorder; IVMP, high dose intravenous methyilprednisolone; LL, lower limbs; LY, lymphocytes; MN, mononuclear cells; MOG, anti-myelin oligodendrocyte glycoprotein antibodies; n/a, data not available; NS/ONA, anti-neuronal surface/onconeural antibodies; OCB, oligoclonal bands; OCS, oral corticosteroid; ON, optic neuritis; PEX, plasma exchange; PN, polymorphonuclear cells; UL, upper limbs.

^a^ Timeframe between vaccine administration and onset of NMOSD/MOGAD symptoms

^b^ Lesions number, localization (LETM, longitudinally extending transverse myelitis; ST, supratentorial; IT, infratentorial; MCP, middle cerebellar peduncle;) and gadolinium enhancement (Gd+/Gd-)
^c^ CSF WBC and protein levels were expressed with –/+/++/+++ for normal or mildly/moderately/markedly elevated levels, considering as value ranges 0-5, 6-25, 26-100, >100 /μL for WBC and 0-45, 46-150, 151-300, >300 mg/dl for protein levels
^d^ Recovery at last available follow-up
