# Supplementary material for: CNS inflammatory demyelinating events after COVID-19 vaccines: A case series and systematic review
Source: Front Neurol. 2022 Dec 1;13:1018785. doi: 10.3389/fneur.2022.1018785 (PMC9752005; doi:10.3389/fneur.2022.1018785)
Supplement: Supplementary file 5 [file Image_1.pdf]

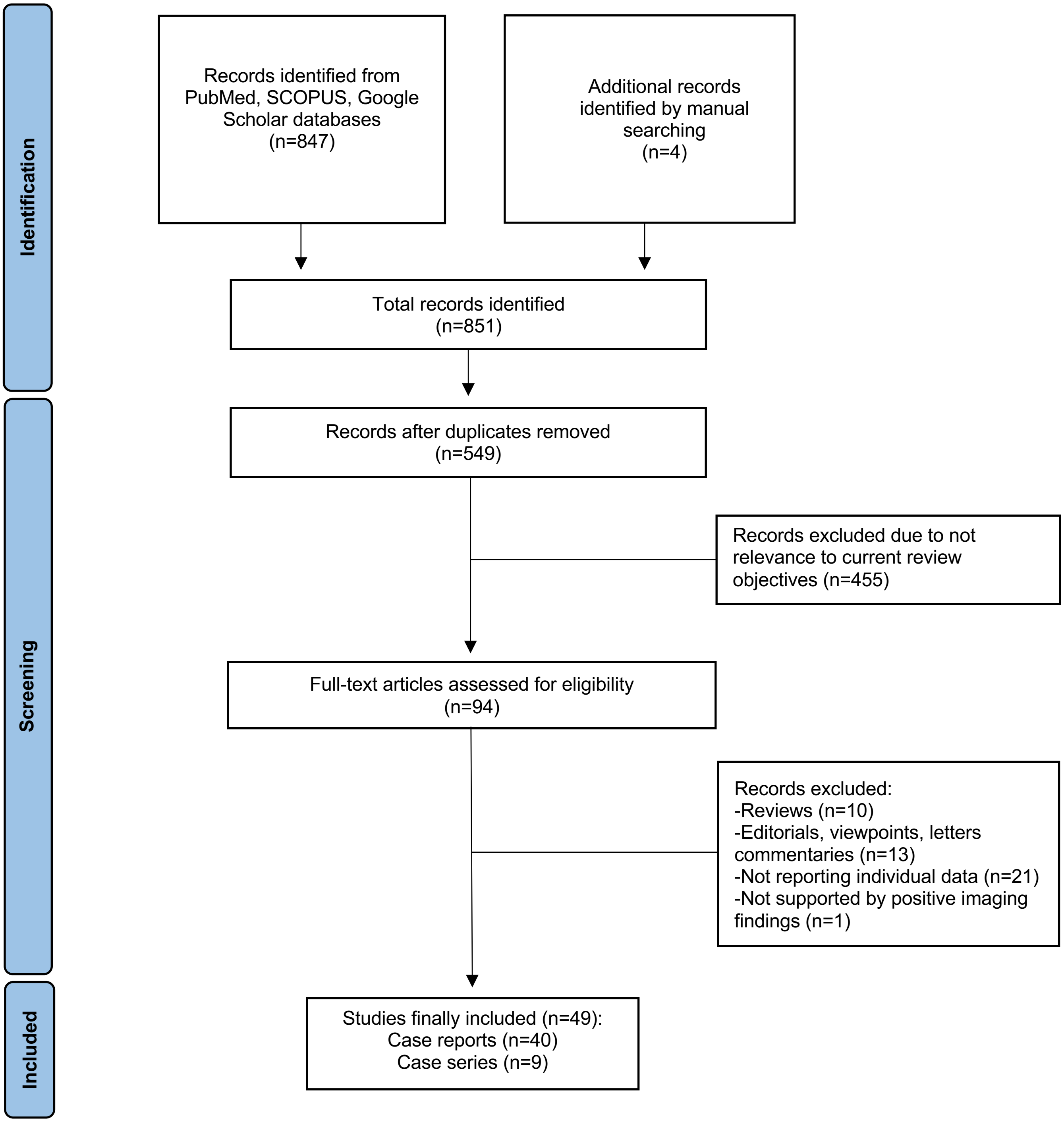

Supplementary figure 1. Preferred reporting items for systematic reviews and meta-analyses (PRISMA) study selection flow diagram
